# Identification of Ideal Allele Combinations for the Adaptation of Spring Barley to Northern Latitudes

**DOI:** 10.3389/fpls.2019.00542

**Published:** 2019-05-03

**Authors:** Magnus Göransson, Jón Hallsteinn Hallsson, Morten Lillemo, Jihad Orabi, Gunter Backes, Ahmed Jahoor, Jónatan Hermannsson, Therese Christerson, Stine Tuvesson, Bo Gertsson, Lars Reitan, Muath Alsheikh, Reino Aikasalo, Mika Isolahti, Merja Veteläinen, Marja Jalli, Lene Krusell, Rasmus L. Hjortshøj, Birger Eriksen, Therése Bengtsson

**Affiliations:** ^1^Faculty of Agricultural and Environmental Sciences, Agricultural University of Iceland, Reykjavik, Iceland; ^2^Department of Plant Sciences, Norwegian University of Life Sciences, Ås, Norway; ^3^Nordic Seed A/S, Odder, Denmark; ^4^Faculty of Organic Agricultural Sciences, Kassel University, Witzenhausen, Germany; ^5^Department of Plant Breeding, The Swedish University of Agricultural Sciences, Alnarp, Sweden; ^6^Lantmännen Lantbruk, Svalöv, Sweden; ^7^Graminor, Hamar, Norway; ^8^Boreal Plant Breeding Ltd., Jokioinen, Finland; ^9^Natural Resources Institute Finland (Luke), Jokioinen, Finland; ^10^Sejet Plant Breeding, Horsens, Denmark

**Keywords:** earliness, GWAS, *Hordeum vulgare*, maturity, plant breeding, plant height, QTL

## Abstract

The northwards expansion of barley production requires adaptation to longer days, lower temperatures and stronger winds during the growing season. We have screened 169 lines of the current barley breeding gene pool in the Nordic region with regards to heading, maturity, height, and lodging under different environmental conditions in nineteen field trials over 3 years at eight locations in northern and central Europe. Through a genome-wide association scan we have linked phenotypic differences observed in multi-environment field trials (MET) to single nucleotide polymorphisms (SNP). We have identified an allele combination, only occurring among a few Icelandic lines, that affects heat sum to maturity and requires 214 growing degree days (GDD) less heat sum to maturity than the most common allele combination in the Nordic spring barley gene pool. This allele combination is beneficial in a cold environment, where autumn frost can destroy a late maturing harvest. Despite decades of intense breeding efforts relying heavily on the same germplasm, our results show that there still exists considerable variation within the current breeding gene pool and we identify ideal allele combinations for regional adaptation, which can facilitate the expansion of cereal cultivation even further northwards.

## Introduction

Climate change has begun to negatively affect yield of cereal crops (Lesk et al., 2016) and is predicted to cause even further yield losses in many of the low latitude grain producing regions of the world (Dai, 2011; Rosenzweig et al., 2014). At the same time global demand for cereals keeps growing (Tilman et al., 2011) caused by both population increase (Gerland et al., 2014) and altered consumption patterns (Kastner et al., 2012). The Nordic region is unique from an agricultural perspective with its relatively mild climate for its northern latitude and a long photoperiod during the growth season (Nurminiemi et al., 1996). Barley (*Hordeum vulgare* L.) is, alongside wheat, one of the dominating cereal crops in the Nordic region^1^, used primarily for feed and malt but with a growing demand for human consumption (Baik and Ullrich, 2008; Baik, 2016). Recent breeding efforts have paved the way for a more reliable barley harvest in the northern marginal area (Lillemo et al., 2010; Hilmarsson et al., 2017), where early flowering and the ability to reach maturity at low temperatures are key components to secure a high and stable yield at high latitudes (Nurminiemi et al., 1996; Hilmarsson et al., 2017). Events of strong winds and heavy precipitation are likely to increase in frequency due to global warming (Coumou and Rahmstorf, 2012). Hence, resistance to lodging and straw breaking are important traits. The heat wave in Scandinavia in the summer of 2018, which led to considerable yield losses, further stresses the importance to address a more volatile and unpredictable future climate. Here, early developing cultivars, which need less time in the field and are thus exposed to potentially damaging weather for a shorter time, could play a role in mitigating the risks. Better understanding of the genetics underlying these traits will enable breeders to produce locally adapted high yielding cultivars for the Nordic and sub-arctic region, further expanding the current cultivation area northwards.

Timing of flowering through seasonal cues, such as day length and temperature, is a key element for reproductive success (Andrés and Coupland, 2012). Earliness is a complex trait where genetic variation can greatly alter the plants response to photoperiod and temperature (Cockram et al., 2007; Blümel et al., 2015). In its region of origin, barley germinates in the fall and stays in the vegetative phase during the cool and humid winter season; increased day length in the spring triggers the onset of flowering and the plants mature at the start of the dry summer period ensuring a period of dormancy for the seeds during the hot and dry summer (Lister et al., 2009). Consistent with its importance for the plant’s survival the response to changes in the photoperiod is controlled by several well conserved genes (Blümel et al., 2015). Among those is the *Ppd-H1* gene, located on chromosome 2H, whose wild type function is to promote flowering under long day conditions (Turner et al., 2005; Jones et al., 2008; Loscos et al., 2014). With the expansion of barley northward with the spread of agriculture, a recessive *ppd-H1* allele with delayed flowering was favored (Jones et al., 2008). This recessive allele helped the spring barley utilize the summer season in the northern latitudes by a less strong up-regulation of the *HvFT1* gene than with the wild type *Ppd-H1* allele (Hemming et al., 2008). Lister et al. (2009) found a latitudinal increase in the prevalence of this recessive allele in historical cultivars and landraces from Europe. Another important gene for the regulation of flowering is *HvCO1*, a gene acting in parallel with *Ppd-H1*, whose overexpression leads to up-regulation of the *HvFT1* gene, which in turn leads to flowering (Campoli et al., 2012; Loscos et al., 2014). *HvCO2* on chromosome 6H is a paralog to *HvCO1* (Campoli et al., 2012). *HvFT1* has alleles with copy number variation (CNV) which have been associated with early flowering in spring barley, a phenotype first discovered in the Finnish cultivar Tammi (Nitcher et al., 2013; Loscos et al., 2014). *HvFT1* has two paralogs: *Ppd-H2* (synonym *HvFT3*) which promotes spikelet initiation (Mulki et al., 2018) and *HvCEN*, which has been shown to affect flowering time and has a latitudinal specific distribution of alleles suggesting adaptive function in the northwards range expansion of barley (Comadran et al., 2012). The *HvCEN* (syn. *eps2S* or *eam6*; Comadran et al., 2012; Alqudah et al., 2016) locus inhibits flowering and is located in the centromeric region of chromosome 2H (Comadran et al., 2012). *HvCEN* has been shown to have a mutant allele that, contrary to the wild type allele, does not inhibit flowering in spring barley and interact with *HvFT1* (Loscos et al., 2014). Another *FT*-like gene is HvFT4 on the short arm of chromosome 2H which is a temperature responsive gene with increased expression in high temperature (Ford et al., 2016). *HvELF3* (syn. *Mat-a* or *Eam8*) on chromosome 1H is a homolog of *Arabidopsis thaliana* gene *ELF3* (Zakhrabekova et al., 2012). The dominant *HvELF3* allele delays flowering in long day conditions while several of the recessive alleles provide day length neutrality which leads to early flowering in both long-day and short-day conditions (Faure et al., 2012). One recessive allele (*mat-a.8*) is the result of an induced mutation in the cultivar Bonus and was released 1960 with the cultivar Mari (Gustafsson et al., 1971; Lundqvist, 2009), the name describing its main characteristics (from Latin for *ma*tura = early and *ri*gida = stiff) (Gustafsson et al., 1971). The day length neutrality associated with the *HvELF3* polymorphism has been proposed to enable cultivation of barley as far north as Iceland, as well as enabling the spread of barley to high altitude regions near the equator (Faure et al., 2012; Zakhrabekova et al., 2012). *Vrn-H1* (*HvAP1*) on chromosome 5H is involved in vernalization requirement and interacts with *Vrn-H2* on chromosome 4H. A wild type recessive *vrn-H1* and a functional *Vrn-H2* always result in a winter growth habit (Karsai et al., 2005; Loscos et al., 2014). Several alleles of *Vrn-H1* exists, resulting in a spring growth habit or a facultative growth habit in lines with a spring allele in *Vrn-H1* or lines where *Vrn-H2* is deleted (Loscos et al., 2014).

In addition to the importance of the correct timing of flowering, plant height matters as demonstrated when dwarfing genes were introduced into wheat in the green revolution (Peng et al., 1999). Intensive cereal cultivation is today dependent on semi-dwarf cultivars (Kuczyńska et al., 2013) since short and strong stems help the plants withstand wind, prevent lodging and can positively affect the harvest index (Hay, 1995). Studies have shown that height reduction is the result of either reduced hormone expression or hormone insensitivity (Dockter et al., 2014), and that two of the main factors regulating plant height are the plant hormones brassinosteroids (BRs) and gibberellic acids (GAs) (Marzec and Alqudah, 2018). BRs are also known to affect traits such as tiller number and grain size in rice (Zhang et al., 2014). In barley, height is controlled by dwarfing and semi-dwarfing genes as well as other genes affecting plant height (Wang et al., 2014). The dwarfing genes are not useful in breeding as they are linked to reduced vigor and yield (Wang et al., 2014). Instead, semi-dwarfing genes have been widely employed in modern barley breeding (Kuczyńska et al., 2013; Wang et al., 2014), these include semi-brachytic 1 (*uzu1*) (Chono et al., 2003), *semi-dwarf 1* (*sdw1*/*denso*) (Jia et al., 2009), *breviaristatum-e* (*ari-e*) (Liu et al., 2014), and short culm 1 (*hcm1*) (Wang et al., 2014). The *uzu1* and *sdw1*/*denso* genes are both located close to the centromere on chromosomal arm 3HL with the *sdw1*/*denso* gene located more distally from the centromere. The *ari-e* locus is located on chromosomal arm 5HL and the *hcm1* gene is located on chromosomal arm 2HL (Wang et al., 2014). Modern European barley cultivars generally depend on the *sdw1/denso* locus as their source of semi-dwarfing (Kuczyńska et al., 2013). Plants carrying the semi-dwarf allele of the *sdw1/denso* locus can be identified morphologically by having a prostrate growth habit in their juvenile stage, whereas plants carrying the dominant allele have an erect juvenile growth habit (Kuczyńska et al., 2013).

High-throughput genotyping has developed as a feasible alternative to traditional genotyping with molecular markers, such as AFLPs. The high-throughput method utilizes single nucleotide polymorphisms (SNP) spread across the genome at an even distribution (Comadran et al., 2012). The development of high-throughput SNP-panels enables a genomic resolution not easily obtained by other marker types. This improved coverage has opened up the possibility to perform genome wide association scans (GWAS) on a variety of agricultural traits (see e.g., Waugh et al., 2014).

Linkage disequilibrium (LD) is the non-random co-segregation of alleles at two loci (Flint-Garcia et al., 2003). LD is generally higher in self-pollinating crops than in out-breeding species and is higher in homogenous than in diverse populations (Flint-Garcia et al., 2003). In association mapping, LD affects the number of markers needed as well as the resolution obtained in the associations (Rafalski, 2002). In self-pollinating crops with very high LD the resolution gets lower as associated markers may be located far away from the responsible locus (Malysheva-Otto et al., 2006). In contrast, when LD is low, the resolution is high as the distance between associated marker and the gene of interest will be short (Remington et al., 2001). A recent study of the same Nordic population as used here confirmed the average LD to be in the range 0–4 cM but with large variations in different chromosomal regions and varying among the population structure groups (Bengtsson et al., 2017).

Better understanding of the allelic diversity in the Nordic breeding material will enable the application of marker-assisted selection for beneficial allele combinations and speed up the breeding process. Detailed understanding of loci controlling earliness and straw stability will enable fine-tuning of cultivars better adapted to northern latitudes. We screened a panel of 169 barley lines from the Nordic breeding pool at eight locations for the traits heading day, maturity day and straw stability with the aim of performing a genome-wide association analysis to identify loci responsible for the quantitative traits earliness and straw stability in multiple environments.

## Materials and Methods

### Plant Material, Test Locations, and Phenotyping

A panel of 169 spring barley lines, representing the Nordic breeding gene pool, were included in the study, selected by each of six different Nordic barley breeding entities (Supplementary Table S1). The main aim of the selection process was to maximize diversity for pathogen resistance, earliness and straw quality, among advanced cultivars and breeding lines. Out of the 169 lines – 124 two-rowed and 45 six-rowed – 58 lines were of Danish origin (all two-rowed), 30 Swedish (28 two-rowed and 2 six-rowed), 30 Norwegian (3 two-rowed and 27 six-rowed), 29 Finnish (all two-rowed), 21 Icelandic (5 two-rowed and 16 six-rowed), and one from the United Kingdom (two-rowed). The two panels used in this study, one consisting of all 169 lines and one with only the 124 two-rowed lines, are referred to as PPP169 and PPP124, respectively.

**FIGURE 1:**
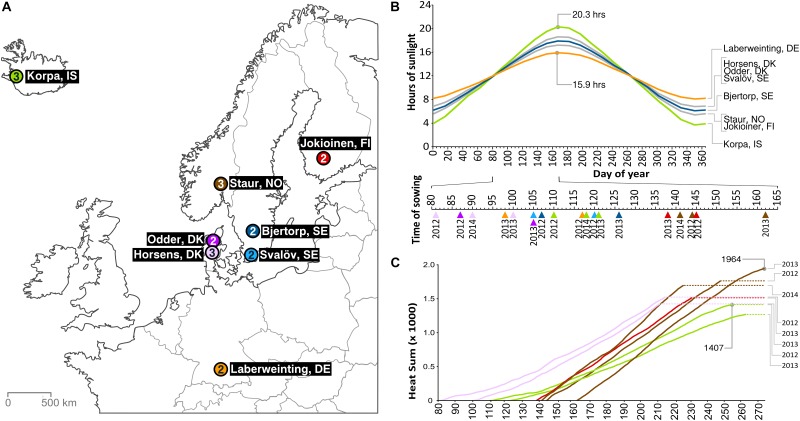
An overview of the multi-environment field trials (MET). **(A)** Field tests were performed at eight different locations in six different countries, ranging from Laberweinting, Germany in the south to Korpa, Iceland in the north, for up to 3 years, resulting in 19 distinct environments. The number of years is shown inside circles. **(B)** The different day lengths between test locations, with the respective sowing days of the trials. **(C)** The accumulated heat sum (°C) from sowing day until maturity of the latest maturing line in the eight trials where maturity was screened. The highest and lowest value are shown in the graph (at Korpa, IS 2013 not all lines reached maturity).

Multi-environment field trials (MET) were performed in eight locations for 2–3 years, resulting in a maximum of 19 distinct environments (Figure 1A and Supplementary Table S1), ranging from Laberweinting, Germany in the south (48°48′6″N) to Korpa, Iceland in the north (64°08′56″N) and Jokioinen, Finland in the east (23°29′54″E) to Korpa in the west (21°45′03″W). Within the environments there is great variation in hours of sunlight during the growth period (Figure 1B) and the heat sum available (Figure 1C). The field trials were set up with up to three replications in an alpha lattice design. Plot size varied between locations from row sowings up to 10 m^2^ field plots. Lodging and straw breaking were not recorded in row sowings, since it could have yielded a different result compared with field plots.

Plants were phenotyped for early spring growth in four environments (measured as height in all lines when the first lines entered growth stage 31, 32, and 34: Ht31, Ht32, and Ht34, respectively) (Zadoks et al., 1974). The measurement was conducted from soil level up to a cardboard plate laid on top of the plants in the plot, as an estimator of the height. Heading day (HD) was recorded in 17 environments as number of days from sowing until half of the spike was visible in 50% of the plants in each plot (stage 53; Zadoks et al., 1974). Maturity day (MD) was recorded in 7 environments as number of days from sowing until the peduncle below the spike turned yellow (approximate growth stage 89; Zadoks et al., 1974). Grain filling period (GFP) was calculated as the period from HD to MD (7 environments). Heat sums for HD (HSHD), MD (HSMD), and GFP (HSGFP) were calculated by adding the maximum daily temperature with the minimum daily temperature and dividing them by 2, then adding the sums for each day of the respective period giving the growing degree-days (GDD), with 0°C used as base line temperature, meaning only sums above 0°C were added. Straw length (StL) was measured upon maturity from soil level to below the spike in cm (11 environments). Lodging (Ld), recorded in 6 environments and straw breaking (SB), recorded in 5 environments were recorded on a scale from 1 to 9 where 1 means no lodging/straw breaking, 5 means 50% lodging/straw breaking and 9 means 100% lodging/straw breaking.

### DNA Extraction and Genotyping

DNA was extracted using a standard CTAB protocol (Cetyl Trimethyl Ammonium Bromide) with DNA extraction kits as described earlier by Orabi et al. (2014). All lines were genotyped with the barley 9K iSelect SNP chip which contains 7842 SNP markers (Comadran et al., 2012). Genotyping was performed by TraitGenetics (Gatersleben, Germany).

6208 SNP markers were polymorphic >5% level and their physical position (in base pairs) on the barley reference genome (Mascher et al., 2017) were retrieved using the online tool BARLEYMAP^2^ (Cantalapiedra et al., 2015).

### Descriptive Statistics

All traits were continuous except for the discrete character row type. For the continuous characters, a normal distribution was expected. To check for deviations from the expected normal distribution, data for all trials and traits were plotted in distribution plots using Excel Add-In XLSTAT v. 19.2. Pearson correlations were calculated to describe the relationships between traits and between field trials using Microsoft Excel 2016.

Descriptive statistics for all environments, separately and combined, were computed with the psych software package v. 1.8.12 (Revelle, 2018) using the R software (R Development Core Team, 2017), this included number of observations (n), mean, standard deviation (sd), median, median absolute deviation (mad), minimum (min), maximum (max), range, skew, kurtosis, and standard error (se).

### Analysis of Variance (ANOVA)

To evaluate the relative contributions of genotype, environment, and genotype by environment interactions in the data set, each trait was analyzed with mixed linear modeling using PROC MIXED in SAS v. 9.4 (SAS Institute Inc.). This initial analysis assumed genotypes, environments, and the genotype by environment interactions to be fixed effects and replications and blocks within replications of the individual trials as random effects.

Best linear unbiased estimates (BLUEs) to be used in the GWAS analyses were calculated using the lmer function in the “lme4” R package (Bates et al., 2015), assuming all effects, except the genotypic effects to be random. Phenotypic data across years were estimated as:

$${\text{y}}_{\text{ijk}}=\;\text{μ}+G_{\text{i}}+en_{\text{j}}+r_{(j)k}+e_{\text{ijk},}$$

where *y*_ijk_ is the *k*th observation of the ith genotype in the jth environment, μ is the common intercept, *G_i_* is the effect of the *i*th genotype, *en_j_* is the effect of the *j*th environment, *r_(j)k_* is the effect of the *k*th replication in environment *j*, and *e_ijk_* is the corresponding error.

### Population Structure

Population structure was evaluated with the software STRUCTURE v.2.3.4 (Falush et al., 2007) and by principal component analysis (PCA) using GenAlEx v.6.5.0.1 (Peakall and Smouse, 2006; Peakall and Smouse, 2012; as in Bengtsson et al., 2017).

For the STRUCTURE analysis, the SNP genotype data was run 10 times with a burn-in period of 9999 followed by 9999 iterations from K = 1 to K = 12. To identify the optimal number of genetic clusters (subpopulations), ΔK values were calculated as proposed by Evanno et al. (2005) using STRUCTURE HARVESTER v. 0.6.94 (accessed 25 Nov. 2015)^3^. The STRUCTURE analysis was previously run for the PPP169 panel in Bengtsson et al. (2017), but here an identical analysis for the PPP124 panel was run. An analysis of molecular variance (AMOVA) was run in GenAlEx to check for variance among the STRUCTURE groups.

### Genome-Wide Association Analysis

Genome-wide association analysis was performed using TASSEL v. 5.2.31 (Bradbury et al., 2007) and using the R package, Genome Association and Prediction Integrated Tool (GAPIT v. 3.0) (Lipka et al., 2012). The hapmap file of the SNP markers was filtered to exclude unsuccessful marker assays, monomorphic markers, and rare alleles (with less than 5% occurrence in the population). Unmapped SNP markers were assigned to an artificial chromosome to capture any associations with these markers.

After filtering 5710 SNP markers remained in the analysis of the PPP169 panel, or 73% of the total number of 7864 SNPs. When the PPP124 panel was filtered to remove markers below 5% polymorphism, 5037 SNP markers remained. Kinship matrices were constructed based on the filtered set of markers using the scaled identity-by-state (IBS) method (Zhang et al., 2010) for both panels. The respective kinship matrices were then used in subsequent mixed linear model (MLM) analyses (Yu et al., 2006; Zhang et al., 2010). To select the correct model and account for population structure we performed four associations using TASSEL: (1) General linear model (GLM) analysis without including population structure in the model; (2) Efficient mixed model association (EMMA) using the kinship matrix with *Q*-values from STRUCTURE K = 2; (3) EMMA using kinship matrix with eigenvalues from the PCA analysis; and (4) EMMA using only the kinship matrix. In addition, the models in TASSEL were compared with the following models using GAPIT v. 3.0: (5) MLM using the van Raden kinship; and (6) MLM using the van Raden kinship with eigenvalues from the PCA analysis. The significant allelic effect estimate is given in relation to the minor allele in the GAPIT output. The MLM was run as optimum level and the P3D (estimated once) variance component estimation. Quantile-quantile (Q-Q) plots were created by comparing expected and observed chi-square values and Manhattan plots showing positions of associated markers across the genome were constructed for each trait using the R package CMplot^4^. Quantile-quantile plots (Q-Q plots) were plotted to assess the goodness of fit of the model for each trait. Large deviations from the expected distribution mean that the model does not fit the data. The models were evaluated trait-wise by comparing the Q-Q plots and checking the narrow sense heritability values, where a higher heritability indicated that the model had a higher predictive value.

GWAS was performed trait-wise on the calculated BLUEs from all trials with the PPP169 panel, as well as on the PPP124 panel which includes only the two-rowed lines. A threshold value was calculated to estimate a significance level for the association analysis. The Bonferroni method based on the total number of markers for each panel and a significance level of 0.05 gave a -log_10_(p) = 5.00 for the PPP124 panel and -log_10_(p) = 5.06 for the PPP169 panel. This is a very stringent method (Gupta et al., 2014) considering that many of the markers are strongly linked. Thus, a suggestive threshold, earlier published by Duggal et al. (2008), allowing for one false positive per genome scan was estimated by dividing 1 by the number of markers for each panel. This resulted in a suggestive threshold of -log_10_(p) = 3.70 for PPP124 and -log_10_(p) = 3.76 for PPP169, which is considered as the significance threshold for the marker-trait associations identified in this study. Both thresholds are indicated in the Manhattan plots.

The length of the quantitative trait loci (QTL) were decided by calculating the LD between the most significant markers at each intra-chromosomal locus using the TASSEL software. The *r*^2^-values earlier reported for each chromosome and each panel (Bengtsson et al., 2017, Supplementary Tables S3) were used as threshold values for determining whether a QTL should be regarded as distinct or not.

### Allele Frequencies and Combinations

Allele combinations were constructed for the traits Ht34, HSHD, HSMD, StL, and SB in the PPP169 panel. The most significantly marker-trait associated SNP marker was used for each QTL and for practical reasons, the total combination of SNPs used to construct the allele combinations was limited to three. The effect of each allele combination with at least five observations (lines) was calculated based on BLUE values and the significance of the effects was tested using the lm () function in R. Allele frequencies were calculated for the SNP markers used in construction of the allele combinations, as well as previously described SNP markers for earliness traits (Comadran et al., 2012; Maurer et al., 2015).

## Results

### Descriptive Statistics and Distributions

Summary statistics were calculated for all nineteen environments, individually and combined (Supplementary Tables S2, S3), and frequency distributions plotted for all phenotypic traits (Supplementary Figure S1). For early vigor (Ht31, Ht32, Ht34), the distributions were generally right-skewed indicating mostly late genotypes in the two panels, with a few very early developing lines. StL, SB, and Ld had normal distributions in both panels. For earliness traits (HD, HSHD, MD, HSMD, GFP, and HSGFP) the distributions were left-skewed, indicating a few early lines with the majority being later developing in both panels. The earliest genotypes were all six rowed, hence were not represented in the PPP124 panel.

### Analysis of Variance and Correlations

For all traits in both panels the genotype, environment, and genotype by environment interaction were significant (*p* < 0.05) except for environment effect in Ht32 in the PPP124 panel (Supplementary Table S4). The results showed that the effect of genotype by far outweighed the genotype by environment interaction for all traits. Pearson correlations performed on overall means for all 12 traits (Table 1) showed that the earliness traits (HD, HSHD, MD, HSMD, GFP, and HSGFP) were all positively correlated (*p* < 0.01), but with negative correlations to the straw properties and the early vigor (StL, SB, Ld, Ht31, Ht32, and Ht34), these were in turn positively intercorrelated.

**Table 1:** Pearson pairwise correlations of overall means for days from sowing to heading (HD), accumulated heat sum from sowing to heading (HSHD), days from sowing to maturity (MD), accumulated heat sum from sowing to maturity (HSMD), grain filling period (registered as the number of days between heading and maturity) (GFP), and the accumulated heat sum in the grain filling period (HSGFP), early vigor (measured as height at growth stage 31, 32, and 34 (Zadoks et al., 1974) (Ht31, Ht32, and Ht34), straw length (StL), straw breaking (SB), and lodging (Ld) for both panel PPP124 and PPP169.

	HD	HSHD	MD	HSMD	GFP	HSGFP	Ht31	Ht32	Ht34	StL	SB
***PPP124***		
HSHD	0.990										
MD	0.865	0.861									
HSMD	0.871	0.867	0.997								
GFP	0.552	0.524	0.870	0.860							
HSGFP	0.449	0.413	0.769	0.770	0.906						
Ht31	–0.694	–0.698	–0.802	–0.798	–0.676	–0.598					
Ht32	–0.705	–0.704	–0.803	–0.800	–0.665	–0.598	0.957				
Ht34	–0.742	–0.741	–0.828	–0.823	–0.672	–0.595	0.948	0.968			
StL	–0.393	–0.416	–0.459	–0.450	–0.357	–0.251	0.537	0.585	0.639		
SB	–0.458	–0.455	–0.558	–0.558	–0.492	–0.435	0.469	0.462	0.503	0.289	
Ld	–0.376	–0.359	–0.433	–0.437	–0.368	–0.338	0.417	0.460	0.509	0.532	0.550
***PPP169***		
HSHD	0.995										
MD	0.930	0.932									
HSMD	0.930	0.932	0.998								
GFP	0.496	0.510	0.780	0.776							
HSGFP	0.587	0.584	0.833	0.839	0.971						
Ht31	–0.811	–0.802	–0.804	–0.807	–0.519	–0.601					
Ht32	–0.835	–0.826	–0.829	–0.830	–0.538	–0.618	0.980				
Ht34	–0.869	–0.862	–0.869	–0.868	–0.575	–0.648	0.962	0.981			
StL	–0.379	–0.394	–0.409	–0.406	–0.321	–0.317	0.534	0.570	0.607		
SB	–0.730	–0.736	–0.747	–0.748	–0.523	–0.570	0.707	0.714	0.753	0.542	
Ld	–0.329	–0.324	–0.382	–0.383	–0.344	–0.372	0.342	0.388	0.427	0.566	0.467


*All correlations were significant (p ≤ 0.01).*

Pearson correlations performed trait-wise between trials (Supplementary Table S5) showed significant correlation between trials for earliness traits and early vigor. Subsequently, these traits were analyzed as means of all trials in the GWAS analyses. More variation was found for straw properties, where single trials were not correlated with the rest. In these cases, trials which had significant correlation were analyzed as means, whereas trials without significant correlation were analyzed separately.

### Population Structure

The STRUCTURE analysis for the PPP169 panel analyzed in Bengtsson et al. (2017) gave a maximum ΔK value at K = 2, where K1 comprised two-rowed lines from southern regions (mainly Finland, southern Sweden, and Denmark), K2 comprised the six-rowed lines and the admixed group was comprised of two-rowed lines from Norway, Iceland and northern Sweden. Here, STRUCTURE analysis of the PPP124 panel revealed the most variation for two subpopulations and AMOVA showed 38% of the genetic variation explained between K groups. If the two STRUCTURE groups were further subdivided according to Tondelli et al. (2013) with an admixed group with less than 0.7 proportion of the genetic variation assigned to K1 or K2, respectively, these three groups explain 35% of the total variation. For practical purposes, the admixed genotypes were assigned to groups, either north-western or south-eastern, based on knowledge of breeding entity.

### Genome-Wide Association Analysis

GWAS was run for 13 and 12 traits for the PPP169 and PPP124 panels, respectively. The best model, of the six models evaluated, was selected for each trait following the criteria mentioned in material and methods (Supplementary Table S6). For all but two traits MLM with the van Raden kinship matrix, was used. For Ld in both panels, MLM with van Raden kinship matrix and eigenvalues from the PCA were used to account for population structure. Results from analyses using MLM van Raden in GAPIT were reported, due to the resulting associations, where GAPIT in a few cases (e.g., for spike type) yielded more peaks that passed the significance threshold and could be explained by known loci for the respective traits.

**FIGURE 2:**
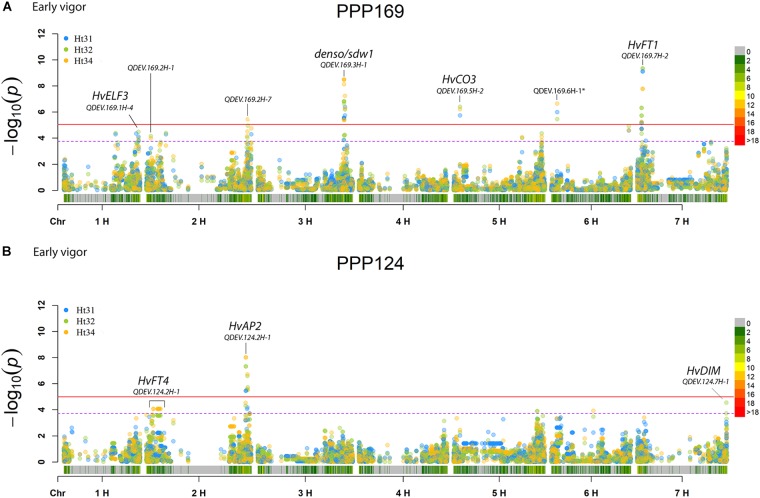
GWAS results for the traits early spring growth stages 31, 32, and 34 (Zadoks et al., 1974), for panels **(A)** PPP169 and **(B)** PPP124, respectively. The Manhattan plots show significant associations (Bonferroni threshold in red, and the suggestive threshold in purple) between trait and marker, the x-axis shows the physical distance over all seven barley chromosomes. The bar under the x-axis shows the SNP distribution on each chromosome, where 0 ->18 depicts SNP density (the number of SNPs per 1 Mbp bin). The non-significant and significant associations are displayed as open and solid filled circles, respectively. The significance of the associations at values between these two extremes are displayed as circles with varying amounts of fill. Relevant QTL names and putative loci are presented in the figure. Note that the ^∗^ indicates a SNP marker located at 3H position 105 cM using the POPSEQ 2017 reference map (http://floresta.eead.csic.es/barleymap/).

**FIGURE 3:**
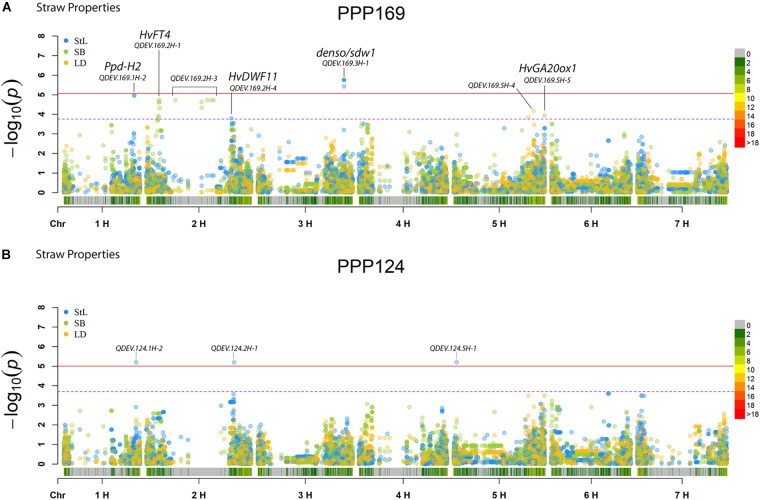
GWAS results for the traits straw length, straw breaking and lodging for panels **(A)** PPP169 and **(B)** PPP124, respectively. The Manhattan plots show significant associations (Bonferroni threshold in red, and the suggestive threshold in purple) between trait and marker, the x-axis shows the physical distance over all seven barley chromosomes. The bar under the x-axis shows the SNP distribution on each chromosome, where 0 ->18 depicts SNP density (the number of SNPs per 1 Mbp bin). The non-significant and significant associations are displayed as open and solid filled circles, respectively. The significance of the associations at values between these two extremes are displayed as circles with varying amounts of fill. The most significant values, located at the top, are displayed as solid filled dots. The significance of the associations at values between these two extremes is indicated by all other dots with varying amounts of fill. Relevant QTL names and putative loci are presented in the figure.

**FIGURE 4:**
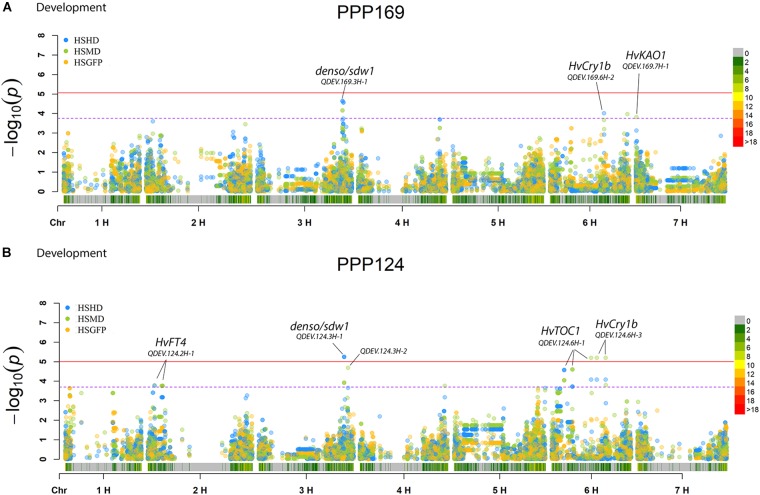
GWAS results for the traits heat sum heading day, heat sum maturity, and heat sum grain filling period for panels **(A)** PPP169 and **(B)** PPP124, respectively. The Manhattan plots show significant associations (Bonferroni threshold in red, and the suggestive threshold in purple) between trait and marker, the x-axis shows the physical distance over all seven barley chromosomes. The bar under the x-axis shows the SNP distribution on each chromosome, where 0 ->18 depicts SNP density (the number of SNPs per 1 Mbp bin). The non-significant and significant associations are displayed as open and solid filled circles, respectively. The significance of the associations at values between these two extremes are displayed as circles with varying amounts of fill. The most significant values, located at the top, are displayed as solid filled dots. The significance of the associations at values between these two extremes is indicated by all other dots with varying amounts of fill. Relevant QTL names and putative loci are presented in the figure.

**FIGURE 5:**
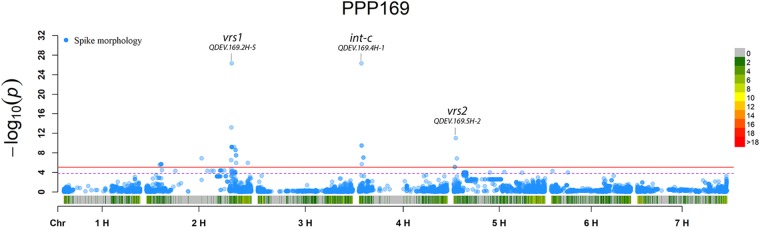
GWAS results for spike morphology for panel PPP169. The Manhattan plots show significant associations (Bonferroni threshold in red, and the suggestive threshold in purple) between trait and marker, the x-axis shows the physical distance over all seven barley chromosomes. The bar under the x-axis shows the SNP distribution on each chromosome, where 0 ->18 depicts SNP density (the number of SNPs per 1 Mbp bin). The non-significant and significant associations are displayed as open and solid filled circles, respectively. The significance of the associations at values between these two extremes are displayed as circles with varying amounts of fill. The most significant values, located at the top, are displayed as solid filled dots. The significance of the associations at values between these two extremes is indicated by all other dots with varying amounts of fill. Relevant QTL names and putative loci are presented in the figure.

In total, for all 12 traits analyzed (excluding spike morphology), 108 significant markers with known genetic position were found with 50 and 45 markers unique for the PPP169 and PPP124 panels, respectively (Supplementary Tables S7, S8). In total 23 and 11 QTL were found in the PPP169 and PPP124 panels, respectively. GWAS results are presented for nine out of twelve traits analyzed, that is for early spring growth stages 31, 32, and 34 (Figure 2), StL, straw breaking and lodging (Figure 3), and heat sum heading, heat sum maturity, and heat sum GFP (Figure 4). Manhattan plots of spike morphology are shown for the PPP169 panel (Figure 5).

### Allelic Diversity and Allele Combinations

Observed allelic diversity for selected SNP markers from the GWAS analyses, and for a set of SNP markers that have previously been associated with flowering genes (Comadran et al., 2012; Maurer et al., 2015), showed different patterns of polymorphisms between the two geographic groups. Several loci were effectively fixed in the south-eastern lines, for example markers nearby or in the *Ppd-H1, HvCO1, HvCO3, HvFT1*, and *denso/sdw1* loci (Supplementary Figure S2). In contrast, alleles for markers nearby the *Vrn-H1* locus were fixed in the north-western lines (Supplementary Figure S2).

**FIGURE 6:**
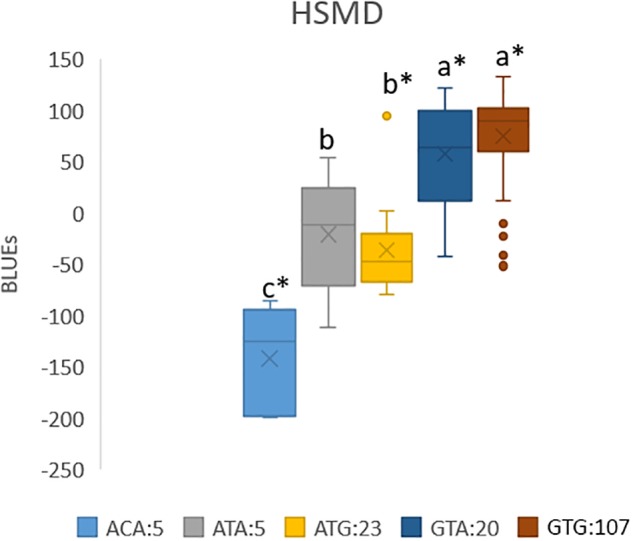
Boxplot of allele combinations showing BLUEs for heat sum to maturity (HSMD) (°C) in the PPP169 panel. Allele combinations were based on three significant markers that passed the suggestive threshold value in the GWAS analysis. The effect of each allele combination for combinations with at least five observations (lines) was calculated based on BLUE values and the significance of the effects was tested using the lm () function in R. ^∗^ marks significant combinations (*p* ≤ 0.05). Allele combinations followed by the same lower case letter do not differ statistically.

Allele combinations, with significance levels for the effects, were constructed for the traits Ht34, HSHD, HSMD, StL, and SB in the PPP169 panel (Figure 6 and Supplementary Figure S3).

## Discussion

### Latitudinal Adaptation of Earliness in Nordic Spring Barley

The barley lines analyzed here could be split in two based on origin, that is into a north-western group and a south-eastern group. The south-eastern group comprised 110 genotypes from breeders in Denmark, Finland, and southern Sweden. The north-western group comprised 58 genotypes from breeders in northern Sweden, Norway, and Iceland. The single genotype from the United Kingdom was not included in the geographical grouping. Despite the north-western group including fewer lines, more diversity was observed there, possibly due to the fact that six-rowed lines were almost exclusively found in this group. When looking only at the PPP124 panel (all two-rows), the pattern was less clear.

A few of the well-known earliness loci had fixed alleles in the two-rowed lines, for example markers in or nearby the *Ppd-H1* and *HvCEN* locus, which could explain why these loci were not detected in the GWAS (Supplementary Figure S2). This is in accordance with previous findings that the wild type *Ppd-H1* and *HvCEN* alleles are fixed in European two-rowed spring barley (Tondelli et al., 2013). *HvCEN* has also been reported as the locus most strongly associated with latitudinal effect among 19 tested flowering associated loci in a European landrace collection (Russell et al., 2016).

Three chromosomal regions have earlier been shown to be of importance in the regulation of flowering in barley; namely chromosomal arm 1HL (*HvELF3* and *Ppd-H2)*, the short and long arm of chromosome 2H (*Ppd-H1* and *HvCEN)*, and chromosomal arm 7HS (*HvFT1* and *HvCO1)* (Loscos et al., 2014). Here we report associations on 1HL for early vigor in the PPP169 panel, but not in the PPP124 panel, with QDEV.169.1H-4 located only 4 Mbp (2.6 cM) from the *HvELF3* locus (Figure 2A). QDEV.169.1H-4 showed diversity in the south-eastern two-rowed lines but had fixed alleles in the north-western two-rowed lines indicating that this locus might have an associated adaptive advantage with increasing latitude.

The strongest association for early vigor in the PPP169 panel (QDEV.169.7H-2) was found on chromosomal arm 7HS. The two most significant markers (both showing an identical pattern) at the 7H-2 QTL, 12_30894, and 12_30895, were both located within the *HvFT1* locus (Supplementary Table S7). The pattern of allelic diversity reflected the geographical origin where both alleles were present among the north-western lines, whereas in the south-eastern lines the allele was fixed (Supplementary Figure S2). Although, the *HvCO1* gene could not be detected in the GWAS analysis, the allele frequency for the known *HvCO1* marker, BK_03 (Comadran et al., 2012), showed a similar pattern as that seen for QDEV.169.7H-2.

Another QTL with strong association (QDEV.169.3H-1) to early vigor, heading, and maturity was detected on chromosomal arm 3HL nearby the known flowering and semi-dwarf locus, *denso/sdw1*, in the PPP169 panel (Figure 2, 4). The QDEV.169.3H-1 had a too low allele frequency in the PPP124 panel (<0.05 MAF) to be detected in the GWAS. The *denso*/*sdw1* locus has been speculated to have an effect on both earliness and height (Kuczyńska et al., 2014), and the fixation of QDEV.169.3H-1 in the two-rowed lines might reflect the historical breeding focus to shorten culm length in Nordic two-rowed lines (Dockter and Hansson, 2015).

Two QTL, QDEV.124.2H-1, and QDEV.124.7H-1, were associated with early vigor exclusively in the PPP124 panel (Figure 2B). QDEV.124.2H-1 included all significant markers detected on chromosome 2H in the PPP124 panel and was further confirmed by the identical allelic pattern observed for these markers. The most significant marker in QDEV.124.2H-1 with a position on 2HL is in close vicinity of *HvAP2*, previously shown to influence tiller number and plant height (Alqudah et al., 2016; Neumann et al., 2017).

For heading (PPP169/PPP124) and maturity (PPP124) we found a QTL located nearby the locus *HvCry1b* (Figure 4), which plays a role in the regulation of seed dormancy (Barrero et al., 2014), and has been reported as a putative heading associated gene (Alqudah et al., 2016).

When the most significantly associated markers for each trait were combined into allele combinations, we identified combinations with strong effect on earliness traits (Figure 6 and Supplementary Figure S3). Especially noteworthy is the allele combination ACA which has a heat sum requirement 214 GDD below the most common allele combinations (GTG and GTA) in the Nordic barley gene pool (Figure 6). The ACA combination only occurred in five of the Icelandic lines (both two-rowed and six-rowed). In average, over all trials where maturity was scored, the difference in heat sum requirement to maturity equals 13.5 days shorter growth season. However, in the cool Icelandic conditions, the difference equals 19.4 days less from sowing to maturity compared with the most common allele combinations. In the sub-arctic environment, where harvest is typically done in September, this time can be the difference between a harvested mature crop and a crop destroyed by autumn frost and/or storms. The finding that the ACA allele combination was only found among Icelandic lines highlights the importance of selecting breeding lines in the target environment.

### Phytohormone-Related Genes Associated With Straw Properties

For StL, we report a QTL (QDEV.169.3H-1) near the *denso/sdw1* locus on chromosome 3H in the PPP169 panel (Figure 3A), with an effect of 9 cm. The GA20 oxidase gene (*Hv20ox2*), involved in biosynthesis of gibberellic acid (GA), has been identified as a candidate for the *denso*/*sdw1* gene which could explain its effect on plant height (Jia et al., 2009). This QTL was, except for six lines, fixed among the two-rowed lines and therefore not detected in the GWAS analysis of the PPP124 panel (Figure 3B).

Another significant QTL (QDEV.169.2H-4), also with an effect of 9 cm, was found on 2H in the PPP169 panel. This is located close to the *HvDWF11* locus (Dockter et al., 2014), which has earlier been reported as a brassinosteroid-related gene in rice (Tanabe et al., 2005).

In the PPP124 panel three QTL, located on chromosomes 1H, 2H, and 5H, were found associated with StL (Figure 3B). The peak at 1H (QDEV.124.1H-2) did not correlate with known loci for height, but Alqudah et al. (2016) found a marker only 8kb distally with an effect on tiller number. The additional QTL, QDEV.124.2H-1, and QDEV.124.5H-1, identified for StL in PPP124 were previously reported to be associated with tiller number by Neumann et al. (2017). QDEV.124.5H-1 has been reported to associate with lodging (Tondelli et al., 2013).

Interestingly, for straw breaking, several significant marker-trait associations within QDEV.169.2H-3 were located nearby the region of the gene *HvGID2* (Figure 3; Marzec and Alqudah, 2018). In addition, a QTL, QDEV.169.5H- 5, was found close to the GA20 oxidase gene *HvGA20ox1* (Alqudah et al., 2016) was identified here to associate with straw breaking.

For lodging two QTL were found on 5HL (Figure 3), one of them, QDEV.169.5H-4, was located nearby a previously reported QTL for lodging (Tondelli et al., 2013). The relatively low significance of the associations with lodging and straw breaking could be explained by the difficulty in scoring these traits as we observed very little lodging or straw breaking the first 2 years. In year three, the nitrogen level was doubled in Denmark and Iceland, to promote lodging, with some success in Iceland but with less success in Denmark.

There was a general difference in the statistical strength of the associations between traits. Associations with earliness traits were weaker than associations with row type, early spring growth, and height. Row type had by far the strongest association. Early spring growth, measured as height of the foliage when plants had reached growth stages 31, 32, and 34 (Zadoks et al., 1974) was the second most significantly associated trait after row type. This trait showed a very high correlation across locations and years, had a high heritability, and therefore potentially a smaller number of controlling loci compared with the flowering pathway. As earliness is known to be controlled by a relatively large and intricate network of loci (Blümel et al., 2015) whereas height is controlled by few loci (Kuczyńska et al., 2013), this suggests that simple inherited traits controlled by few loci were more easily detected than complex traits controlled by multiple loci. These findings are therefore a validation of the GWAS model used.

## Conclusion

Although, most lines in our study showed a low degree of straw breaking we identified one allele combination, GGA, with a significantly higher rate of straw breaking (Supplementary Figure S3). This allele combination could be used to actively select against weak straw in the Nordic breeding programs.

The BLUE distributions showed a considerable difference between the two panels, with the PPP169 panel having a greater range of diversity for all traits. This is, at least for the earliness traits and the early vigor, most likely due to a small number of extremely early six-rowed barley lines from Iceland, that all headed earlier than 50 days in the field trials (see Supplementary Figure S1). The low number of these extremely early lines, only 3 such lines were included, made it hard to detect the effects of the underlying loci in the GWAS. Interestingly, the extremely early lines from Iceland, all carried the same allele at markers BK_12, BK_14, BK_15, and BK_16, all located within the *Ppd-H1* locus, different from the rest of the Nordic material. However, this allele combination was also found in the single two-rowed line of intermediate earliness from the United Kingdom. The fixed allele combination among the four *Ppd-H1* associated markers is therefore insufficient to explain the extreme earliness observed in the Icelandic material, and no other loci are found in the GWAS that could on their own explain the extreme earliness. Evidence does, however, suggest that a polymorphism at the markers 12_30894 and 12_30895, both located within the *HvFT1* locus, found in the Icelandic lines and not in the previously mentioned United Kingdom line might at least partly explain this observation. To further elucidate the genetics behind the unique agronomic performances of these extremely early lines a segregating multi-parent advanced generation intercross (MAGIC) population has been produced.

We here report the first GWAS of developmental traits focusing exclusively on Nordic spring barley from all five Nordic countries including both two- and six-rowed cultivars. Previous studies have found Nordic barleys to carry allelic diversity in many loci affecting early heading and early maturity (Tondelli et al., 2013; Loscos et al., 2014). This was confirmed in our study. In a few of the known flowering loci the pattern of allelic diversity is clearly different between row types, for example alleles for markers located in the *HvFT1* and *HvCEN* genes are fixed in the two-rowed lines but there is diversity among the six-rowed lines. Based on our results we could identify ideal allele combinations for regional adaptation to the unique day length and climate conditions in the extremely northern latitude, which could help push the margin for barley cultivation both in the north and possibly at other marginal areas.

## Author Contributions

AJ, MV, MA, LR, GB, JO, BG, RH, MG, and BE were involved in the planning and experimental design of the study. LK, RH, RA, MI, MJ, LR, TC, MG, and JH were managing the field experiments and phenotyping in field. JO, GB, and MG were managing the laboratory experiments. TB, MG, JO, ML, GB, and JHH performed data and statistical analyses. MG, TB, JHH, and ML wrote and critically reviewed the manuscript, made the figures and finalized the tables. All authors contributed to the discussion of the results and the editing and approval of the final manuscript.

## Conflict of Interest Statement

JO and AJ were employed by Nordic Seed A/S, Denmark. TC, ST, and BG were employed by Lantmännen Lantbruk, Sweden. LR and MA were employed by Graminor, Norway. RA, MI, and MV were employed by Boreal Plant Breeding Ltd., Finland. LK, RH, and BE were employed by Sejet Plant Breeding, Denmark. The remaining authors declare that the research was conducted in the absence of any commercial or financial relationships that could be construed as a potential conflict of interest.

## Footnotes

1 http://faostat3.fao.org/

2 http://floresta.eead.csic.es/barleymap/

3 http://taylor0.biology.ucla.edu/structureHarvester/

4 https://github.com/YinLiLin/R-Cmplot
