# Supplementary material for: Identification of Ideal Allele Combinations for the Adaptation of Spring Barley to Northern Latitudes
Source: Front Plant Sci. 2019 May 3;10:542. doi: 10.3389/fpls.2019.00542 (PMC6510284; doi:10.3389/fpls.2019.00542)
Supplement: Supplementary file 5 [file Table_5.docx]

**Table S5**. Trait-wise correlations of multi-environment field trials for early vigor (measured as height at growth stage 31, 32, and 34 (Zadoks *et al.,* 1974) (Ht31, Ht32, and Ht34), straw length (StL), straw breaking (Sb), lodging (Ld), days from sowing to heading (HD), accumulated heat sum from sowing to heading (HSHD), days from sowing to maturity (MD), accumulated heat sum from sowing to maturity (HSMD), grain filling period (registered as the number of days between heading and maturity) (GFP), and the accumulated heat sum in the grain filling period (HSGFP) for both panel PPP124 and PPP169. Significant levels (p ≤ 0.05) are indicated with a *.

| *StL_PPP169* | *StL_Ko_12* | *StL_Ho_12* | *StL_Ko_13* | *StL_Ho_13* | *StL_Ko_14* | *StL_Bj_12* | *StL_Sv_12* | *StL_St_12* | *StL_St_13* | *Stl_St_14* | *StL_Bo_13* |
| --- | --- | --- | --- | --- | --- | --- | --- | --- | --- | --- | --- |
| StL_Ho_12 | 0.73* |  |  |  |  |  |  |  |  |  |  |
| StL_Ko_13 | 0.94* | 0.75* |  |  |  |  |  |  |  |  |  |
| StL_Ho_13 | 0.68* | 0.81* | 0.71* |  |  |  |  |  |  |  |  |
| StL_Ko_14 | 0.87* | 0.76* | 0.91* | 0.74* |  |  |  |  |  |  |  |
| StL_Bj_12 | 0.51* | 0.51* | 0.51* | 0.49* | 0.49* |  |  |  |  |  |  |
| StL_Sv_12 | 0.14 | 0.11 | 0.15* | 0.08 | 0.10 | 0.21* |  |  |  |  |  |
| StL_St_12 | 0.83* | 0.80* | 0.85* | 0.78* | 0.82* | 0.51* | 0.09 |  |  |  |  |
| StL_St_13 | 0.33* | 0.45* | 0.38* | 0.47* | 0.36* | 0.24* | 0.09 | 0.39* |  |  |  |
| Stl_St_14 | 0.60* | 0.66* | 0.61* | 0.71* | 0.59* | 0.36* | 0.06 | 0.71* | 0.39* |  |  |
| StL_Bo_13 | 0.33* | 0.50* | 0.31* | 0.59* | 0.39* | 0.15* | -0.03 | 0.52* | 0.32* | 0.64* |  |
| StL_Od_13 | 0.36* | 0.64* | 0.35* | 0.62* | 0.45* | 0.35* | 0.11 | 0.55* | 0.32* | 0.58* | 0.66* |

| *StL_PPP124* | *StL_Ko_12* | *StL_Ho_12* | *StL_Ko_13* | *StL_Ho_13* | *StL_Ko_14* | *StL_Bj_12* | *StL_Sv_12* | *StL_St_12* | *StL_St_13* | *Stl_St_14* | *StL_Bo_13* |
| --- | --- | --- | --- | --- | --- | --- | --- | --- | --- | --- | --- |
| StL_Ho_12 | 0.59* |  |  |  |  |  |  |  |  |  |  |
| StL_Ko_13 | 0.82* | 0.71* |  |  |  |  |  |  |  |  |  |
| StL_Ho_13 | 0.58* | 0.78* | 0.65* |  |  |  |  |  |  |  |  |
| StL_Ko_14 | 0.70* | 0.67* | 0.82* | 0.65* |  |  |  |  |  |  |  |
| StL_Bj_12 | 0.31* | 0.34* | 0.33* | 0.38* | 0.32* |  |  |  |  |  |  |
| StL_Sv_12 | 0.08 | -0.05 | 0.05 | 0.00 | -0.02 | 0.15 |  |  |  |  |  |
| StL_St_12 | 0.70* | 0.77* | 0.79* | 0.71* | 0.69* | 0.38* | -0.02 |  |  |  |  |
| StL_St_13 | 0.33* | 0.44* | 0.41* | 0.42* | 0.35* | 0.12 | 0.03 | 0.46* |  |  |  |
| Stl_St_14 | 0.46* | 0.63* | 0.56* | 0.62* | 0.5* | 0.34* | 0.03 | 0.63* | 0.4* |  |  |
| StL_Bo_13 | 0.34* | 0.53* | 0.37* | 0.56* | 0.43* | 0.19* | -0.06 | 0.54* | 0.28* | 0.56* |  |
| StL_Od_13 | 0.53* | 0.68* | 0.57* | 0.65* | 0.57* | 0.38* | 0.09 | 0.69* | 0.29* | 0.51* | 0.57* |

| *Ht31_PPP169* | *Ht31_Ko_12* | *Ht31_Ko_13* | *Ht31_Ho_12* |
| --- | --- | --- | --- |
| Ht31_Ko_13 | 0.87* |  |  |
| Ht31_Ho_12 | 0.79* | 0.76* |  |
| Ht31_Ho_13 | 0.83* | 0.81* | 0.82* |

| *Ht31_PPP124* | *Ht31_Ko_12* | *Ht31_Ko_13* | *Ht31_Ho_12* |
| --- | --- | --- | --- |
| Ht31_Ko_13 | 0.72* |  |  |
| Ht31_Ho_12 | 0.77* | 0.70* |  |
| Ht31_Ho_13 | 0.65* | 0.58* | 0.72* |

| *Ht32_PPP169* | *Ht32_Ko_12* | *Ht32_Ko_13* | *Ht32_Ho_12* |
| --- | --- | --- | --- |
| Ht32_Ko_13 | 0.90* |  |  |
| Ht32_Ho_12 | 0.85* | 0.85* |  |
| Ht32_Ho_13 | 0.90* | 0.91* | 0.91* |

| *Ht32_PPP124* | *Ht32_Ko_12* | *Ht32_Ko_13* | *Ht32_Ho_12* |
| --- | --- | --- | --- |
| Ht32_Ko_13 | 0.80* |  |  |
| Ht32_Ho_12 | 0.78* | 0.83* |  |
| Ht32_Ho_13 | 0.78* | 0.86* | 0.82* |

| *Ht34_PPP169* | *Ht34_Ko_12* | *Ht34_Ko_13* | *Ht34_Ho_12* |
| --- | --- | --- | --- |
| Ht34_Ko_13 | 0.94* |  |  |
| Ht34_Ho_12 | 0.90* | 0.89* |  |
| Ht34_Ho_13 | 0.92* | 0.93* | 0.93* |

| *Ht34_PPP124* | *Ht34_Ko_12* | *Ht34_Ko_13* | *Ht34_Ho_12* |
| --- | --- | --- | --- |
| Ht34_Ko_13 | 0.87* |  |  |
| Ht34_Ho_12 | 0.87* | 0.88* |  |
| Ht34_Ho_13 | 0.83* | 0.89* | 0.89* |

| *Sb_PPP169* | *Sb_Ko_12* | *Sb_Ko_13* | *Sb_Ko_14* | *Sb_Ho_12* |
| --- | --- | --- | --- | --- |
| Sb_Ko_13 | 0.47* |  |  |  |
| Sb_Ko_14 | 0.46* | 0.80* |  |  |
| Sb_Ho_12 | 0.67* | 0.47* | 0.49* |  |
| Sb_Ho_13 | 0.70* | 0.45* | 0.42* | 0.85* |

| *Sb_PPP124* | *Sb_Ko_12* | *Sb_Ko_13* | *Sb_Ko_14* | *Sb_Ho_12* |
| --- | --- | --- | --- | --- |
| Sb_Ko_13 | 0.18* |  |  |  |
| Sb_Ko_14 | 0.11 | 0.74* |  |  |
| Sb_Ho_12 | 0.14* | 0.19* | 0.22* |  |
| Sb_Ho_13 | 0.28* | 0.10 | 0.09 | 0.69* |

| *Ld_PPP169* | *Ld_Ko_12* | *Ld_Ko_13* | *Ld_Ko_14* | *Ld_Ho_14* | *Ld_St_12* |
| --- | --- | --- | --- | --- | --- |
| Ld_Ko_13 | 0.50* |  |  |  |  |
| Ld_Ko_14 | 0.32* | 0.41* |  |  |  |
| Ld_Ho_14 | 0.39* | 0.51* | 0.34* |  |  |
| Ld_St_12 | 0.10 | -0.14 | 0.03 | 0.15* |  |
| LD_St_14 | 0.08 | -0.27* | 0.01 | 0.06 | 0.35* |

| *Ld_PPP124* | *Ld_Ko_12* | *Ld_Ko_13* | *Ld_Ko_14* | *Ld_Ho_14* | *Ld_St_12* |
| --- | --- | --- | --- | --- | --- |
| Ld_Ko_13 | 0.50* |  |  |  |  |
| Ld_Ko_14 | 0.31* | 0.28* |  |  |  |
| Ld_Ho_14 | 0.34* | 0.5* | 0.31* |  |  |
| Ld_St_12 | 0.35* | 0.27* | 0.20* | 0.38* |  |
| Ld_St_14 | 0.16 | -0.08 | 0.25* | 0.23* | 0.19* |

| *HD_PPP169* | *HD_Ko_12* | *HD_Ko_13* | *HD_Ho_12* | *HD_Ho_13* | *HD_Bj_12* | *HD_Bj_13* | *HD_Sv_12* | *HD_Sv_13* | *HD_La_12* | *HD_La_13* | *HD_Od_12* | *HD_Od_13* | *HD_St_12* | *HD_St_13* | *HD_St_14* | *HD_Jo_12* |
| --- | --- | --- | --- | --- | --- | --- | --- | --- | --- | --- | --- | --- | --- | --- | --- | --- |
| HD_Ko_13 | 0.91* |  |  |  |  |  |  |  |  |  |  |  |  |  |  |  |
| HD_Ho_12 | 0.87* | 0.90* |  |  |  |  |  |  |  |  |  |  |  |  |  |  |
| HD_Ho_13 | 0.86* | 0.90* | 0.93* |  |  |  |  |  |  |  |  |  |  |  |  |  |
| HD_Bj_12 | 0.86* | 0.89* | 0.89* | 0.89* |  |  |  |  |  |  |  |  |  |  |  |  |
| HD_Bj_13 | 0.86* | 0.89* | 0.87***** | 0.88* | 0.90* |  |  |  |  |  |  |  |  |  |  |  |
| HD_Sv_12 | 0.81* | 0.88* | 0.93* | 0.88* | 0.87* | 0.86* |  |  |  |  |  |  |  |  |  |  |
| HD_Sv_13 | 0.80* | 0.83* | 0.86* | 0.89***** | 0.86* | 0.84* | 0.81* |  |  |  |  |  |  |  |  |  |
| HD_La_12 | 0.84* | 0.84* | 0.91* | 0.93* | 0.89* | 0.86* | 0.86* | 0.89* |  |  |  |  |  |  |  |  |
| HD_La_13 | 0.81* | 0.84* | 0.90* | 0.91* | 0.87* | 0.85* | 0.87* | 0.93* | 0.92* |  |  |  |  |  |  |  |
| HD_Od_12 | 0.80* | 0.84* | 0.91* | 0.88* | 0.83* | 0.81* | 0.87* | 0.81* | 0.86* | 0.84* |  |  |  |  |  |  |
| HD_Od_13 | 0.85* | 0.88* | 0.93* | 0.93* | 0.87* | 0.86* | 0.88* | 0.87* | 0.92* | 0.91* | 0.93* |  |  |  |  |  |
| HD_St_12 | 0.87* | 0.92* | 0.92* | 0.93* | 0.91* | 0.91* | 0.91* | 0.86* | 0.90* | 0.90* | 0.87* | 0.90* |  |  |  |  |
| HD_St_13 | 0.78* | 0.83* | 0.83* | 0.84* | 0.85* | 0.85* | 0.82* | 0.83* | 0.81* | 0.84* | 0.83* | 0.86* | 0.88* |  |  |  |
| HD_St_14 | 0.82* | 0.83* | 0.85* | 0.89* | 0.86* | 0.85* | 0.80* | 0.87* | 0.88* | 0.88* | 0.82* | 0.89* | 0.89* | 0.89* |  |  |
| HD_Jo_12 | 0.82* | 0.81* | 0.82* | 0.84* | 0.84* | 0.81* | 0.77* | 0.84* | 0.86* | 0.84* | 0.78* | 0.84* | 0.85* | 0.81* | 0.87* |  |
| HD_Jo_13 | 0.79* | 0.78* | 0.78* | 0.81* | 0.80* | 0.76* | 0.73* | 0.81* | 0.80* | 0.82* | 0.79* | 0.84* | 0.81* | 0.86* | 0.87* | 0.86* |

| *HD_PPP124* | *HD_Ko_12* | *HD_Ko_13* | *HD_Ho_12* | *HD_Ho_13* | *HD_Bj_12* | *HD_Bj_13* | *HD_Sv_12* | *HD_Sv_13* | *HD_La_12* | *HD_La_13* | *HD_Od_12* | *HD_Od_13* | *HD_St_12* | *HD_St_13* | *HD_St_14* | *HD_Jo_12* |
| --- | --- | --- | --- | --- | --- | --- | --- | --- | --- | --- | --- | --- | --- | --- | --- | --- |
| HD_Ko_13 | 0.79* |  |  |  |  |  |  |  |  |  |  |  |  |  |  |  |
| HD_Ho_12 | 0.66* | 0.73* |  |  |  |  |  |  |  |  |  |  |  |  |  |  |
| HD_Ho_13 | 0.67* | 0.75* | 0.84* |  |  |  |  |  |  |  |  |  |  |  |  |  |
| HD_Bj_12 | 0.67* | 0.70* | 0.81* | 0.79* |  |  |  |  |  |  |  |  |  |  |  |  |
| HD_Bj_13 | 0.70* | 0.71***** | 0.75* | 0.76* | 0.78* |  |  |  |  |  |  |  |  |  |  |  |
| HD_Sv_12 | 0.52* | 0.61* | 0.81* | 0.72* | 0.70* | 0.64* |  |  |  |  |  |  |  |  |  |  |
| HD_Sv_13 | 0.64* | 0.74* | 0.90* | 0.85* | 0.83* | 0.78* | 0.76* |  |  |  |  |  |  |  |  |  |
| HD_La_12 | 0.62* | 0.66* | 0.86* | 0.83* | 0.82* | 0.74* | 0.75* | 0.86* |  |  |  |  |  |  |  |  |
| HD_La_13 | 0.59* | 0.67***** | 0.89* | 0.84* | 0.81* | 0.74* | 0.79* | 0.88* | 0.88* |  |  |  |  |  |  |  |
| HD_Od_12 | 0.50* | 0.55***** | 0.74* | 0.68* | 0.63* | 0.55* | 0.65* | 0.71* | 0.65* | 0.67* |  |  |  |  |  |  |
| HD_Od_13 | 0.62* | 0.71* | 0.89* | 0.82***** | 0.80* | 0.75* | 0.78* | 0.87* | 0.81* | 0.87* | 0.85* |  |  |  |  |  |
| HD_St_12 | 0.68* | 0.76* | 0.83* | 0.87* | 0.80* | 0.79* | 0.74* | 0.84* | 0.83* | 0.85* | 0.65* | 0.81* |  |  |  |  |
| HD_St_13 | 0.54* | 0.63* | 0.72* | 0.71* | 0.69* | 0.69* | 0.65* | 0.75* | 0.64* | 0.72* | 0.66* | 0.76* | 0.76* |  |  |  |
| HD_St_14 | 0.65* | 0.73* | 0.81* | 0.84* | 0.77* | 0.81* | 0.71* | 0.85* | 0.79* | 0.84* | 0.66* | 0.82* | 0.86* | 0.82* |  |  |
| HD_Jo_12 | 0.65* | 0.68* | 0.76* | 0.76* | 0.76* | 0.73* | 0.66* | 0.79* | 0.77* | 0.78* | 0.59* | 0.72* | 0.78* | 0.69* | 0.75* |  |
| HD_Jo_13 | 0.64* | 0.70* | 0.72* | 0.72* | 0.76* | 0.68* | 0.66* | 0.75* | 0.67* | 0.73* | 0.70* | 0.78* | 0.74* | 0.83* | 0.78* | 0.74* |

| *MD_PPP169* | *MD_Ko_12* | *MD_Ho_12* | *MD_Ho_13* | *MD_St_12* | *MD_St_13* | *MD_St_14* |
| --- | --- | --- | --- | --- | --- | --- |
| MD_Ho_12 | 0.87* |  |  |  |  |  |
| MD_Ho_13 | 0.82* | 0.84* |  |  |  |  |
| MD_St_12 | 0.77* | 0.75* | 0.75* |  |  |  |
| MD_St_13 | 0.85* | 0.85* | 0.86* | 0.83* |  |  |
| MD_St_14 | 0.84* | 0.82* | 0.84* | 0.83* | 0.88* |  |
| MD_Jo_13 | 0.84* | 0.85* | 0.88* | 0.81* | 0.90* | 0.90* |

| *MD_PPP124* | *MD_Ko_12* | *MD_Ho_12* | *MD_Ho_13* | *MD_St_12* | *MD_St_13* | *MD_St_14* |
| --- | --- | --- | --- | --- | --- | --- |
| MD_Ho_12 | 0.78* |  |  |  |  |  |
| MD_Ho_13 | 0.67* | 0.76* |  |  |  |  |
| MD_St_12 | 0.58* | 0.54* | 0.50* |  |  |  |
| MD_St_13 | 0.74* | 0.75* | 0.76* | 0.63* |  |  |
| MD_St_14 | 0.71* | 0.74* | 0.72* | 0.67* | 0.80* |  |
| MD_Jo_13 | 0.70* | 0.76* | 0.80* | 0.63* | 0.84* | 0.82* |

| *GFP_PPP169* | *GFP_Ko_12* | *GFP_Ho_12* | *GFP_Ho_13* | *GFP_St_12* | *GFP_St_13* | *GFP_St_14* |
| --- | --- | --- | --- | --- | --- | --- |
| GFP_Ho_12 | 0.25* |  |  |  |  |  |
| GFP_Ho_13 | 0.38* | 0.30* |  |  |  |  |
| GFP_St_12 | 0.11 | 0.21* | 0.10 |  |  |  |
| GFP_St_13 | 0.69* | 0.05 | 0.32* | 0.11 |  |  |
| GFP_St_14 | 0.64* | 0.12 | 0.25* | 0.18* | 0.63* |  |
| GFP_Jo_13 | 0.69* | 0.03 | 0.36* | 0.13 | 0.77* | 0.65* |

| *GFP_PPP124* | *GFP_Ko_12* | *GFP_Ho_12* | *GFP_Ho_13* | *GFP_St_12* | *GFP_St_13* | *GFP_St_14* |
| --- | --- | --- | --- | --- | --- | --- |
| GFP_Ho_12 | 0.53* |  |  |  |  |  |
| GFP_Ho_13 | 0.38* | 0.32* |  |  |  |  |
| GFP_St_12 | 0.17 | 0.13 | -0.04 |  |  |  |
| GFP_St_13 | 0.57* | 0.44* | 0.38* | 0.18* |  |  |
| GFP_St_14 | 0.54* | 0.42* | 0.25* | 0.36* | 0.49* |  |
| GFP_Jo_13 | 0.58* | 0.38* | 0.36* | 0.14 | 0.63* | 0.55* |

| *HSHD_PPP169* | *HSHD_Ko_12* | *HSHD_Ko_13* | *HSHD_Ho_12* | *HSHD_Ho_13* | *HSHD_Bj_12* | *HSHD_Bj_13* | *HSHD_Sv_12* | *HSHD_Sv_13* | *HSHD_La_12* | *HSHD_La_13* | *HSHD_Od_12* | *HSHD_Od_13* | *HSHD_St_12* | *HSHD_St_13* | *HSHD_St_14* | *HSHD_Jo_12* |
| --- | --- | --- | --- | --- | --- | --- | --- | --- | --- | --- | --- | --- | --- | --- | --- | --- |
| HSHD_Ko_13 | 0.91* |  |  |  |  |  |  |  |  |  |  |  |  |  |  |  |
| HSHD_Ho_12 | 0.87* | 0.89* |  |  |  |  |  |  |  |  |  |  |  |  |  |  |
| HSHD_Ho_13 | 0.86* | 0.89* | 0.93* |  |  |  |  |  |  |  |  |  |  |  |  |  |
| HSHD_Bj_12 | 0.86* | 0.87* | 0.88* | 0.88* |  |  |  |  |  |  |  |  |  |  |  |  |
| HSHD_Bj_13 | 0.86* | 0.88* | 0.86* | 0.87* | 0.89* |  |  |  |  |  |  |  |  |  |  |  |
| HSHD_Sv_12 | 0.81* | 0.87* | 0.92* | 0.87* | 0.86* | 0.86* |  |  |  |  |  |  |  |  |  |  |
| HSHD_Sv_13 | 0.84* | 0.85* | 0.93* | 0.93* | 0.89* | 0.86* | 0.88* |  |  |  |  |  |  |  |  |  |
| HSHD_La_12 | 0.84* | 0.85* | 0.93* | 0.93* | 0.89* | 0.86* | 0.88* | 1.00* |  |  |  |  |  |  |  |  |
| HSHD_La_13 | 0.80* | 0.81* | 0.88* | 0.90* | 0.85* | 0.83* | 0.84* | 0.92* | 0.92* |  |  |  |  |  |  |  |
| HSHD_Od_12 | 0.80* | 0.83* | 0.90* | 0.87* | 0.81* | 0.80* | 0.87* | 0.86* | 0.86* | 0.81* |  |  |  |  |  |  |
| HSHD_Od_13 | 0.85* | 0.89* | 0.94* | 0.92* | 0.88* | 0.86* | 0.90* | 0.92* | 0.92* | 0.90* | 0.93* |  |  |  |  |  |
| HSHD_St_12 | 0.73* | 0.79* | 0.77* | 0.76* | 0.79* | 0.78* | 0.77* | 0.74* | 0.74* | 0.74* | 0.78* | 0.81* |  |  |  |  |
| HSHD_St_13 | 0.76* | 0.79* | 0.81* | 0.80* | 0.82* | 0.81* | 0.79* | 0.77* | 0.77* | 0.80* | 0.79* | 0.83* | 0.88* |  |  |  |
| HSHD_St_14 | 0.78* | 0.80* | 0.81* | 0.82* | 0.82* | 0.80* | 0.78* | 0.81* | 0.81* | 0.82* | 0.80* | 0.85* | 0.86* | 0.88* |  |  |
| HSHD_Jo_12 | 0.72* | 0.77* | 0.75* | 0.77* | 0.78* | 0.75* | 0.72* | 0.76* | 0.76* | 0.77* | 0.76* | 0.80* | 0.84* | 0.83* | 0.86* |  |
| HSHD_Jo_13 | 0.73* | 0.76* | 0.76* | 0.79* | 0.79* | 0.74* | 0.72* | 0.76* | 0.76* | 0.79* | 0.74* | 0.81* | 0.81* | 0.86* | 0.87* | 0.88* |

| *HSHD_PPP124* | *HSHD_Ko_12* | *HSHD_Ko_13* | *HSHD_Ho_12* | *HSHD_Ho_13* | *HSHD_Bj_12* | *HSHD_Bj_13* | *HSHD_Sv_12* | *HSHD_Sv_13* | *HSHD_La_12* | *HSHD_La_13* | *HSHD_Od_12* | *HSHD_Od_13* | *HSHD_St_12* | *HSHD_St_13* | *HSHD_St_14* | *HSHD_Jo_12* |
| --- | --- | --- | --- | --- | --- | --- | --- | --- | --- | --- | --- | --- | --- | --- | --- | --- |
| HSHD_Ko_13 | 0.79* |  |  |  |  |  |  |  |  |  |  |  |  |  |  |  |
| HSHD_Ho_12 | 0.67* | 0.73* |  |  |  |  |  |  |  |  |  |  |  |  |  |  |
| HSHD_Ho_13 | 0.67* | 0.74* | 0.84* |  |  |  |  |  |  |  |  |  |  |  |  |  |
| HSHD_Bj_12 | 0.66* | 0.69* | 0.80* | 0.78* |  |  |  |  |  |  |  |  |  |  |  |  |
| HSHD_Bj_13 | 0.70* | 0.71* | 0.74* | 0.75* | 0.77* |  |  |  |  |  |  |  |  |  |  |  |
| HSHD_Sv_12 | 0.52* | 0.61* | 0.80* | 0.70* | 0.70* | 0.65* |  |  |  |  |  |  |  |  |  |  |
| HSHD_Sv_13 | 0.61* | 0.66* | 0.88* | 0.83* | 0.81* | 0.73* | 0.76* |  |  |  |  |  |  |  |  |  |
| HSHD_La_12 | 0.61* | 0.66* | 0.88* | 0.83* | 0.81* | 0.73* | 0.76* | 1.00* |  |  |  |  |  |  |  |  |
| HSHD_La_13 | 0.58* | 0.63* | 0.84* | 0.82* | 0.78* | 0.73* | 0.76* | 0.86* | 0.86* |  |  |  |  |  |  |  |
| HSHD_Od_12 | 0.48* | 0.53* | 0.72* | 0.67* | 0.61* | 0.53* | 0.65* | 0.64* | 0.64* | 0.61* |  |  |  |  |  |  |
| HSHD_Od_13 | 0.61* | 0.71* | 0.89* | 0.81* | 0.80* | 0.75* | 0.80* | 0.82* | 0.82* | 0.82* | 0.83* |  |  |  |  |  |
| HSHD_St_12 | 0.50* | 0.61* | 0.69* | 0.69* | 0.66* | 0.65* | 0.60* | 0.62* | 0.62* | 0.65* | 0.63* | 0.74* |  |  |  |  |
| HSHD_St_13 | 0.50* | 0.56* | 0.70* | 0.65* | 0.65* | 0.65* | 0.61* | 0.59* | 0.59* | 0.68* | 0.60* | 0.72* | 0.77* |  |  |  |
| HSHD_St_14 | 0.54* | 0.62* | 0.71* | 0.70* | 0.68* | 0.68* | 0.65* | 0.66* | 0.66* | 0.69* | 0.64* | 0.75* | 0.87* | 0.80* |  |  |
| HSHD_Jo_12 | 0.46* | 0.60* | 0.65* | 0.66* | 0.61* | 0.61* | 0.59* | 0.61* | 0.61* | 0.62* | 0.62* | 0.70* | 0.82* | 0.73* | 0.75* |  |
| HSHD_Jo_13 | 0.46* | 0.57* | 0.68* | 0.69* | 0.65* | 0.62* | 0.60* | 0.62* | 0.62* | 0.67* | 0.58* | 0.70* | 0.81* | 0.81* | 0.78* | 0.79* |

| *HSMD_PPP169* | *HSMD_Ko_12* | *HSMD_Ho_12* | *HSMD_Ho_13* | *HSMD_St_12* | *HSMD_St_13* | *HSMD_St_14* |
| --- | --- | --- | --- | --- | --- | --- |
| HSMD_Ho_12 | 0.89* |  |  |  |  |  |
| HSMD_Ho_13 | 0.84* | 0.84* |  |  |  |  |
| HSMD_St_12 | 0.77* | 0.74* | 0.73* |  |  |  |
| HSMD_St_13 | 0.86* | 0.85* | 0.85* | 0.81* |  |  |
| HSMD_St_14 | 0.84* | 0.83* | 0.83* | 0.81* | 0.88* |  |
| HSMD_Jo_13 | 0.86* | 0.85* | 0.87* | 0.79* | 0.89* | 0.90* |

| *HSMD_PPP124* | *HSMD_Ko_12* | *HSMD_Ho_12* | *HSMD_Ho_13* | *HSMD_St_12* | *HSMD_St_13* | *HSMD_St_14* |
| --- | --- | --- | --- | --- | --- | --- |
| HSMD_Ho_12 | 0.80* |  |  |  |  |  |
| HSMD_Ho_13 | 0.73* | 0.77* |  |  |  |  |
| HSMD_St_12 | 0.58* | 0.53* | 0.48* |  |  |  |
| HSMD_St_13 | 0.76* | 0.75* | 0.75* | 0.60* |  |  |
| HSMD_St_14 | 0.74* | 0.74* | 0.72* | 0.64* | 0.80* |  |
| HSMD_Jo_13 | 0.74* | 0.76* | 0.79* | 0.58* | 0.83* | 0.83* |

| *HSGFP_PPP169* | *HSGFP_Ko_12* | *HSGFP_Ho_12* | *HSGFP_Ho_13* | *HSGFP_St_12* | *HSGFP_St_13* | *HSGFP_St_14* |
| --- | --- | --- | --- | --- | --- | --- |
| HSGFP_Ho_12 | 0.53* |  |  |  |  |  |
| HSGFP_Ho_13 | 0.58* | 0.43* |  |  |  |  |
| HSGFP_St_12 | 0.13 | 0.17* | 0.11 |  |  |  |
| HSGFP_St_13 | 0.62* | 0.36* | 0.44* | 0.14 |  |  |
| HSGFP_St_14 | 0.42* | 0.30* | 0.30* | 0.14 | 0.56* |  |
| HSGFP_Jo_13 | 0.65* | 0.34* | 0.59* | 0.14 | 0.69* | 0.45* |

| *HSGFP_PPP124* | *HSGFP_Ko_12* | *HSGFP_Ho_12* | *HSGFP_Ho_13* | *HSGFP_St_12* | *HSGFP_St_13* | *HSGFP_St_14* |
| --- | --- | --- | --- | --- | --- | --- |
| HSGFP_Ho_12 | 0.60* |  |  |  |  |  |
| HSGFP_Ho_13 | 0.53* | 0.48* |  |  |  |  |
| HSGFP_St_12 | 0.11 | 0.10 | 0.03 |  |  |  |
| HSGFP_St_13 | 0.48* | 0.45* | 0.39* | 0.16 |  |  |
| HSGFP_St_14 | 0.35* | 0.37* | 0.24* | 0.14 | 0.52* |  |
| HSGFP_Jo_13 | 0.48* | 0.41* | 0.52* | 0.13 | 0.53* | 0.35* |
