# Supplementary material for: Identification of Ideal Allele Combinations for the Adaptation of Spring Barley to Northern Latitudes
Source: Front Plant Sci. 2019 May 3;10:542. doi: 10.3389/fpls.2019.00542 (PMC6510284; doi:10.3389/fpls.2019.00542)
Supplement: Supplementary file 9 [file Data_Sheet_1.PDF]

Figure S1.

Distributions of phenotypic data for early vigor (measured as height at growth stage 31, 32, and 34 (Zadoks *et al.*, 1974) (Ht31, Ht32, and Ht34), straw length (StL), straw breaking (Sb), lodging (Ld), days from sowing to heading (HD), accumulated heat sum from sowing to heading (HSHD), days from sowing to maturity (MD), accumulated heat sum from sowing to maturity (HSMD), grain filling period (registered as the number of days between heading and maturity) (GFP), and the accumulated heat sum in the grain filling period (HSGFP) for both panel PPP169 (on the left) and PPP124 (on the right).

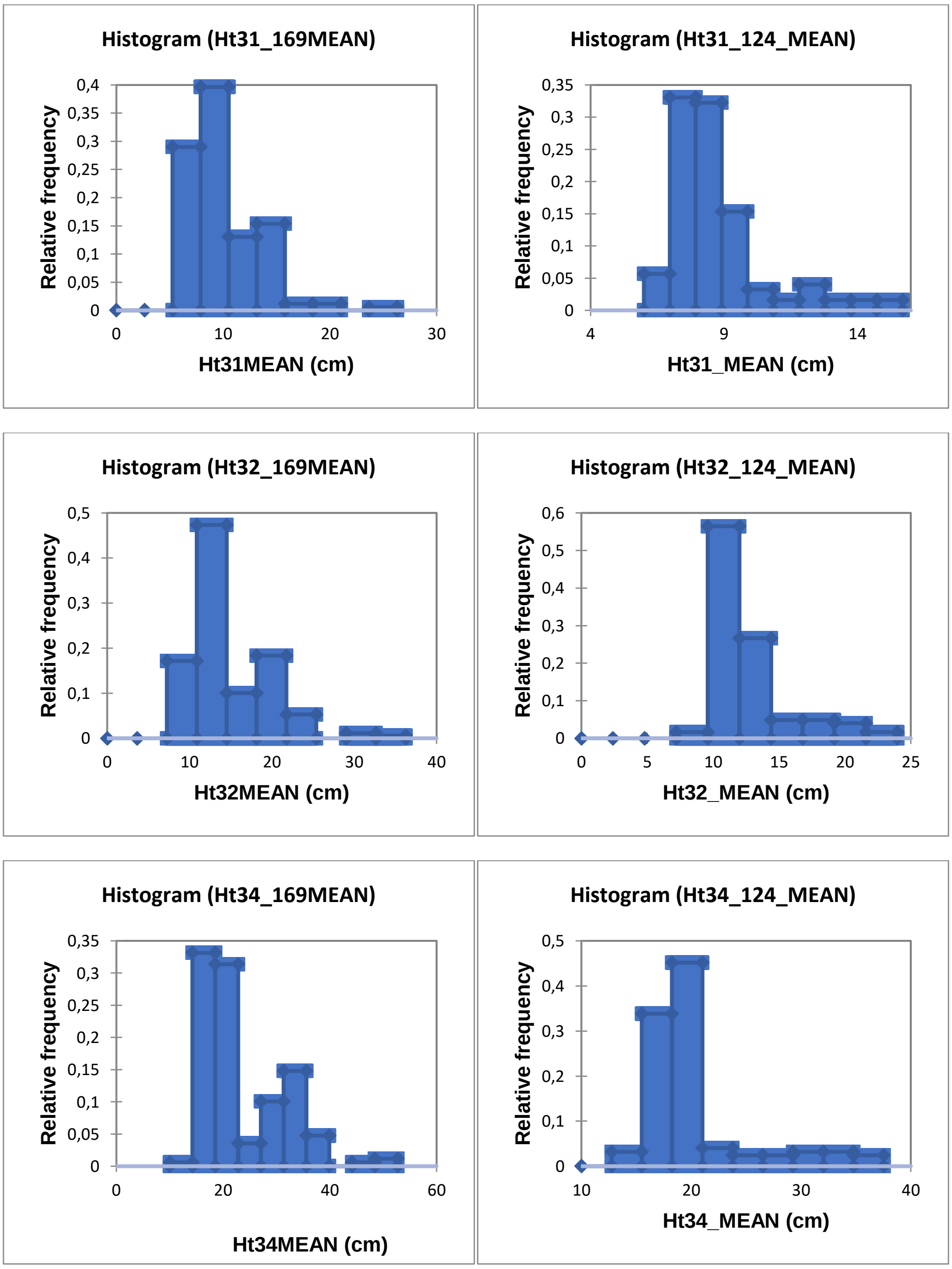

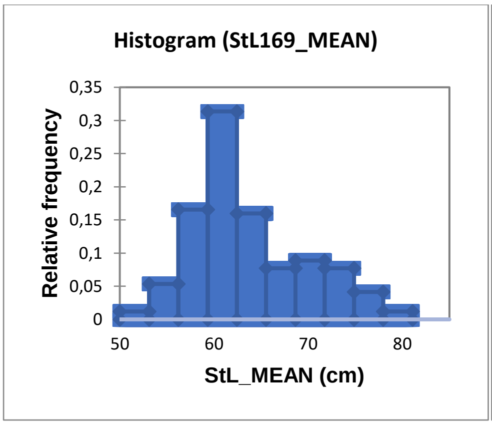
Histogram (StL169_MEAN)
Relative frequency
0,35
0,3
0,25
0,2
0,15
0,1
0,05
0
50
60
70
80
StL_MEAN (cm)

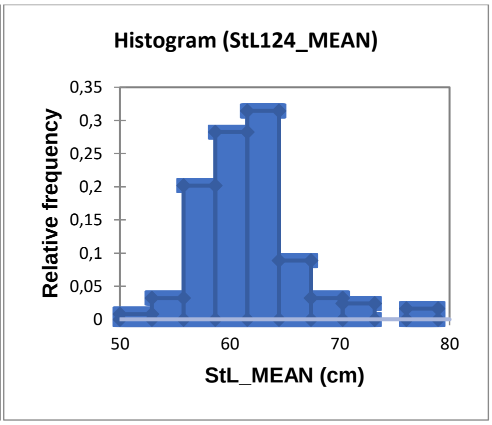
Histogram (StL124_MEAN)
Relative frequency
0,35
0,3
0,25
0,2
0,15
0,1
0,05
0
50
60
70
80
StL_MEAN (cm)

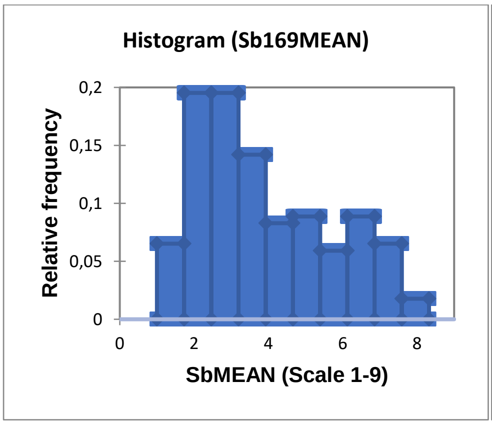
Histogram (Sb169MEAN)
Relative frequency
0,2
0,15
0,1
0,05
0
0
2
4
6
8
SbMEAN (Scale 1-9)

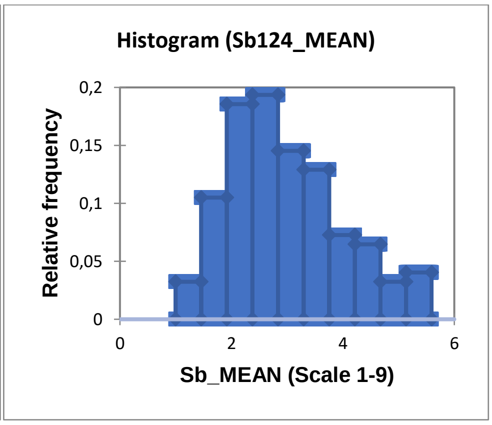
Histogram (Sb124_MEAN)
Relative frequency
0,2
0,15
0,1
0,05
0
0
2
4
6
Sb_MEAN (Scale 1-9)

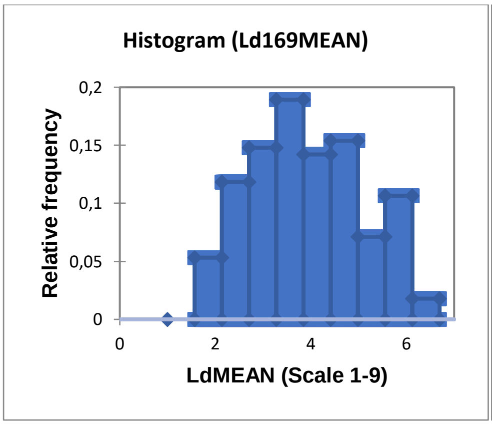
Histogram (Ld169MEAN)
Relative frequency
0,2
0,15
0,1
0,05
0
0
2
4
6
LdMEAN (Scale 1-9)

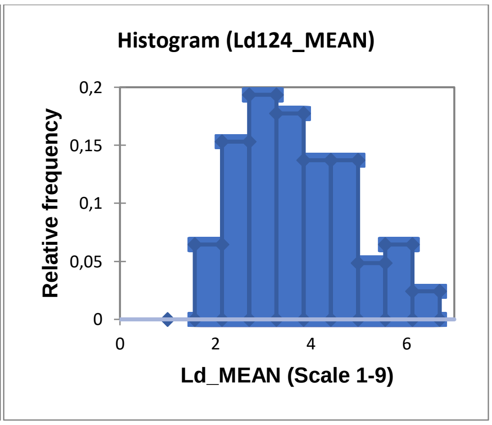
Histogram (Ld124_MEAN)
Relative frequency
0,2
0,15
0,1
0,05
0
0
2
4
6
Ld_MEAN (Scale 1-9)

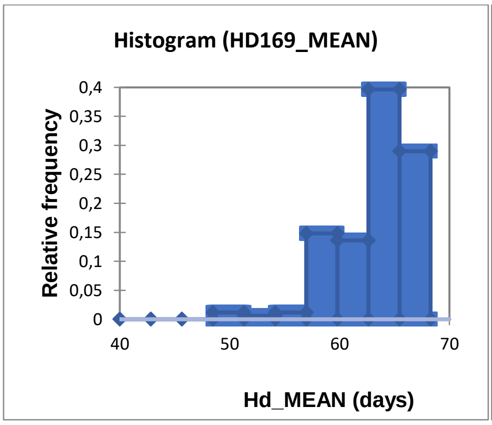
Histogram (HD169_MEAN)
Relative frequency
0,4
0,35
0,3
0,25
0,2
0,15
0,1
0,05
0
40
50
60
70
Hd_MEAN (days)

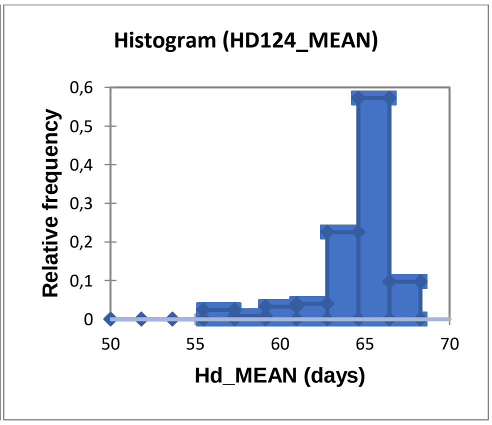
Histogram (HD124_MEAN)
Relative frequency
0,6
0,5
0,4
0,3
0,2
0,1
0
50
55
60
65
70
Hd_MEAN (days)

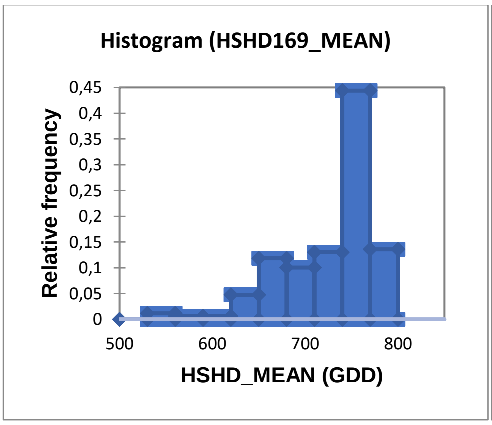
Histogram (HSHD169_MEAN)
Relative frequency
0,45
0,4
0,35
0,3
0,25
0,2
0,15
0,1
0,05
0
500
600
700
800
HSHD_MEAN (GDD)

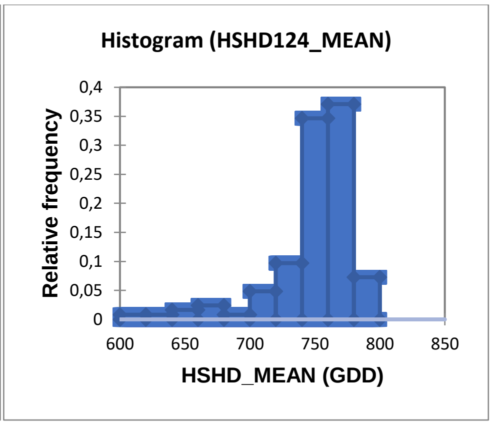
Histogram (HSHD124_MEAN)
Relative frequency
0,4
0,35
0,3
0,25
0,2
0,15
0,1
0,05
0
600
650
700
750
800
850
HSHD_MEAN (GDD)

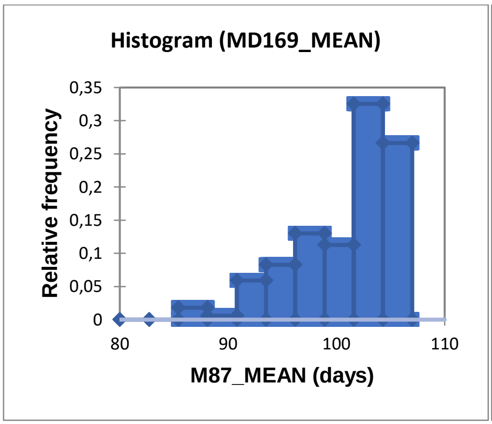
Histogram (MD169_MEAN)
Relative frequency
0,35
0,3
0,25
0,2
0,15
0,1
0,05
0
80
90
100
110
M87_MEAN (days)

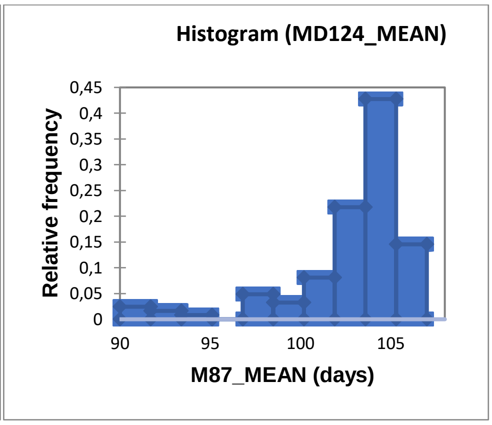
Histogram (MD124_MEAN)
Relative frequency
0,45
0,4
0,35
0,3
0,25
0,2
0,15
0,1
0,05
0
90
95
100
105
M87_MEAN (days)

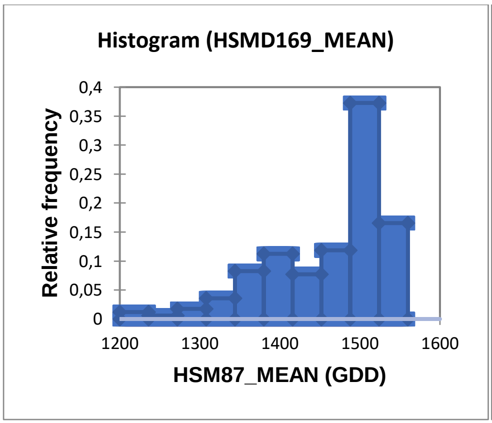
Histogram (HSMD169_MEAN)
Relative frequency
0,4
0,35
0,3
0,25
0,2
0,15
0,1
0,05
0
1200
1300
1400
1500
1600
HSM87_MEAN (GDD)

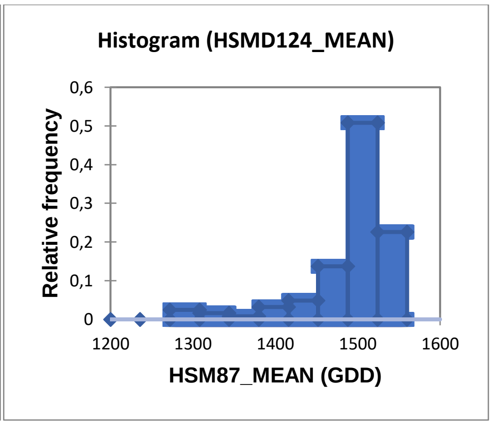
Histogram (HSMD124_MEAN)
Relative frequency
0,6
0,5
0,4
0,3
0,2
0,1
0
1200
1300
1400
1500
1600
HSM87_MEAN (GDD)

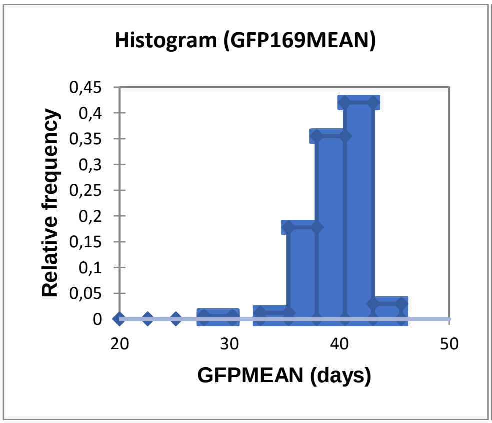
Histogram (GFP169MEAN)
Relative frequency
0,45
0,4
0,35
0,3
0,25
0,2
0,15
0,1
0,05
0
20
30
40
50
GFPMEAN (days)

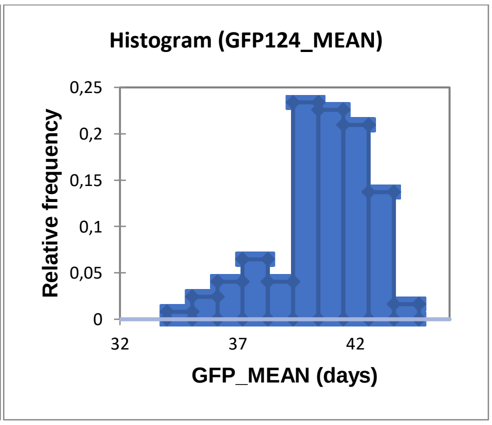
Histogram (GFP124_MEAN)
Relative frequency
0,25
0,2
0,15
0,1
0,05
0
32
37
42
GFP_MEAN (days)

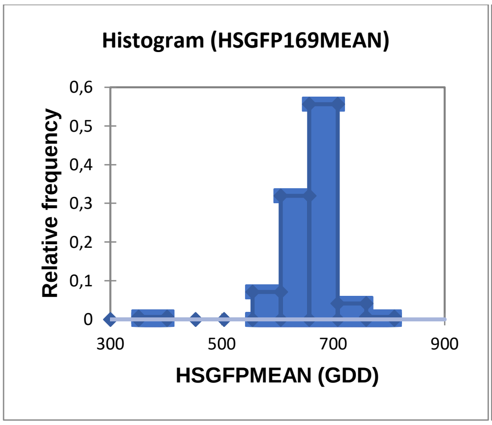
Histogram (HSGFP169MEAN)
Relative frequency
0,6
0,5
0,4
0,3
0,2
0,1
0
300
500
700
900
HSGFPMEAN (GDD)

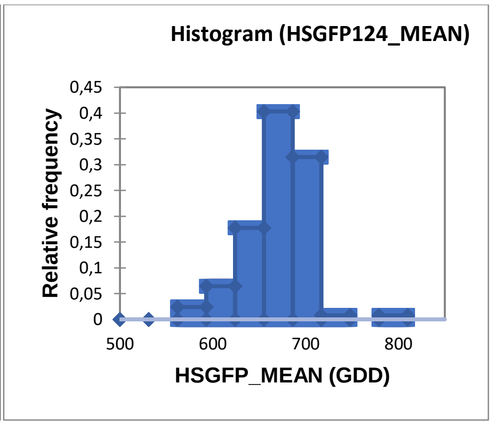
Histogram (HSGFP124_MEAN)
Relative frequency
0,45
0,4
0,35
0,3
0,25
0,2
0,15
0,1
0,05
0
500
600
700
800
HSGFP_MEAN (GDD)
