# Supplementary material for: Identification of Ideal Allele Combinations for the Adaptation of Spring Barley to Northern Latitudes
Source: Front Plant Sci. 2019 May 3;10:542. doi: 10.3389/fpls.2019.00542 (PMC6510284; doi:10.3389/fpls.2019.00542)
Supplement: Supplementary file 10 [file Data_Sheet_2.pdf]

Figure S2. Allele information of the significant SNPs used for construction of allele combinations for the traits: early vigor (measured as height at growth stage 34 (Zadoks *et al.*, 1974) (Ht34), accumulated heat sum from sowing to heading (HSHD), accumulated heat sum from sowing to maturity (HSMD), straw length (StL) and straw breaking (SB), in panel PPP169. Allele information of previously reported SNPs associated with earliness (Comadran *et al.*, 2012; Maurer *et al.*, 2015). The y-axis shows the number of lines carrying the respective allele. The lines are grouped by their origin: north-western (NW) or south-eastern (SE).

Ht34

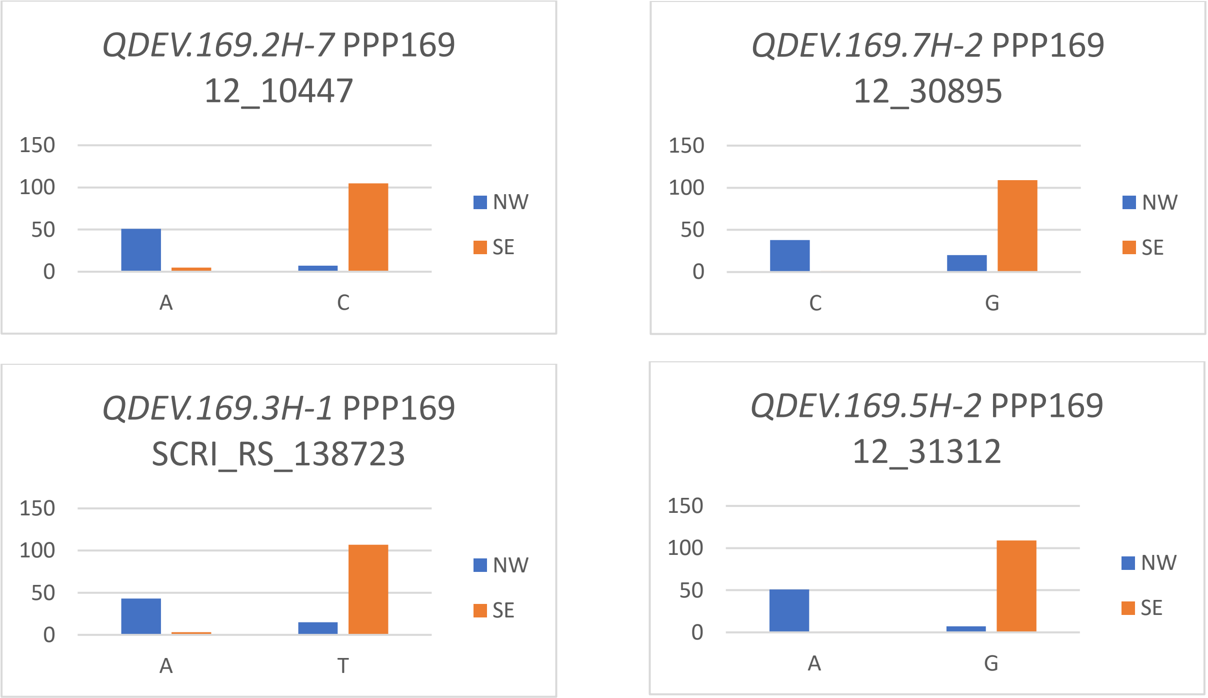

HSHD

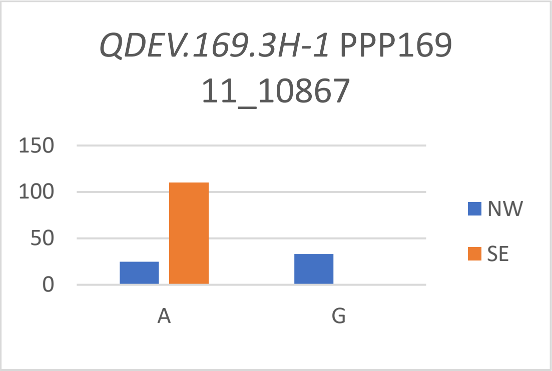

QDEV.169.3H-1 PPP169
11_10867
150
100
50
0
A
G
NW
SE

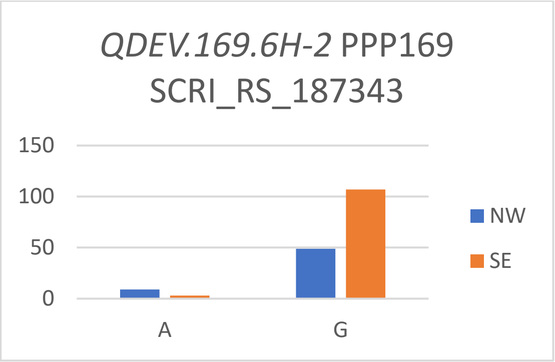

QDEV.169.6H-2 PPP169
SCRI_RS_187343
150
100
50
0
A
G
NW
SE

HSMD

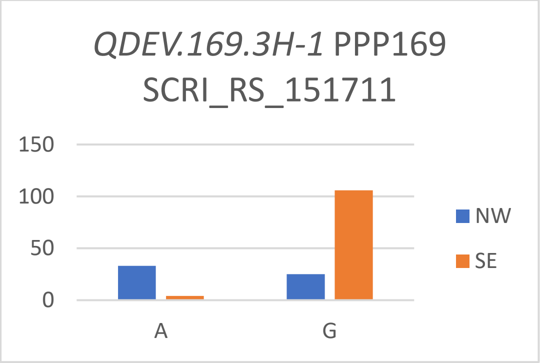

QDEV.169.3H-1 PPP169
SCRI_RS_151711
150
100
50
0
A
G
NW
SE

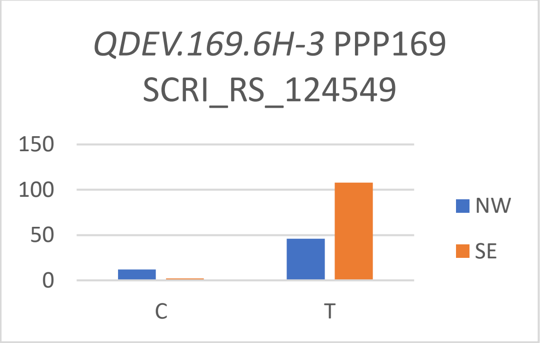

QDEV.169.6H-3 PPP169
SCRI_RS_124549
150
100
50
0
C
T
NW
SE

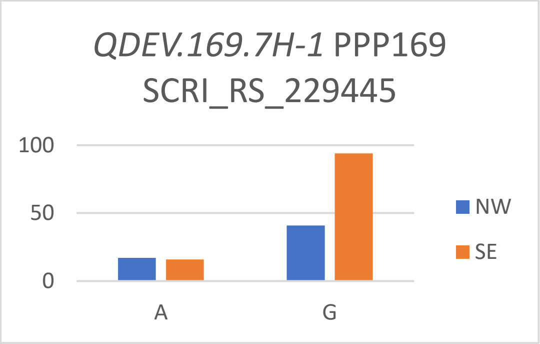

QDEV.169.7H-1 PPP169
SCRI_RS_229445
100
50
0
A
G
NW
SE

SB

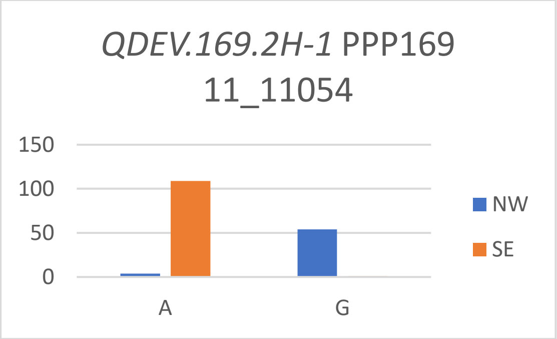
QDEV.169.2H-1 PPP169 11_11054
150
100
50
0
A
G
NW
SE

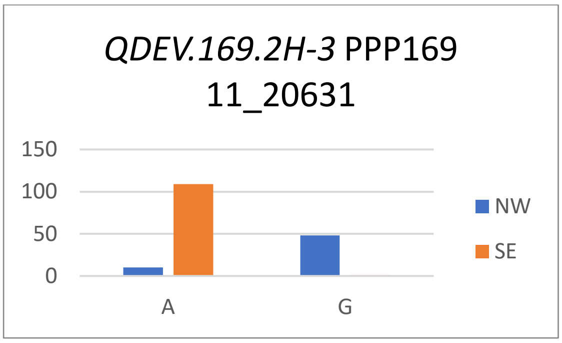
QDEV.169.2H-3 PPP169 11_20631
150
100
50
0
A
G
NW
SE

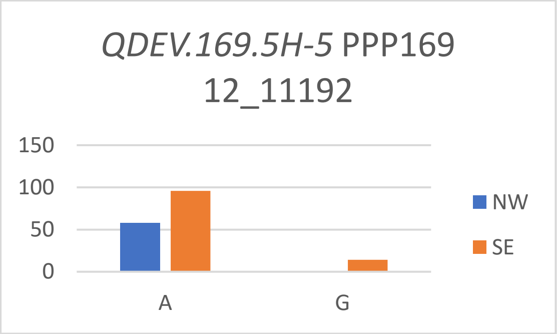
QDEV.169.5H-5 PPP169 12_11192
150
100
50
0
A
G
NW
SE

StL

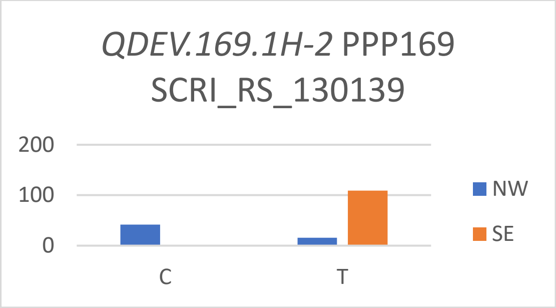
QDEV.169.1H-2 PPP169
SCRI_RS_130139
200
100
0
C
T
NW
SE

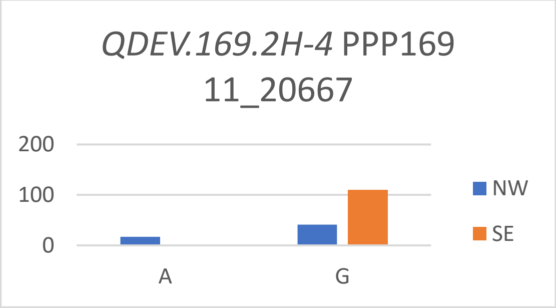
QDEV.169.2H-4 PPP169
11_20667
200
100
0
A
G
NW
SE

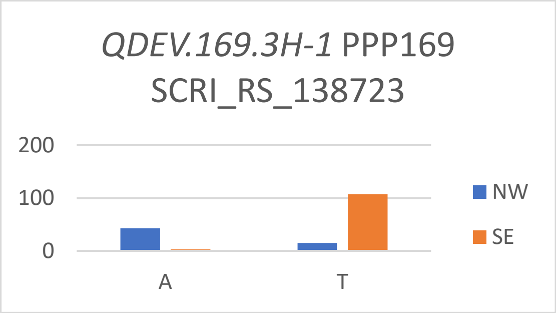
QDEV.169.3H-1 PPP169
SCRI_RS_138723
200
100
0
A
T
NW
SE

Known earliness loci

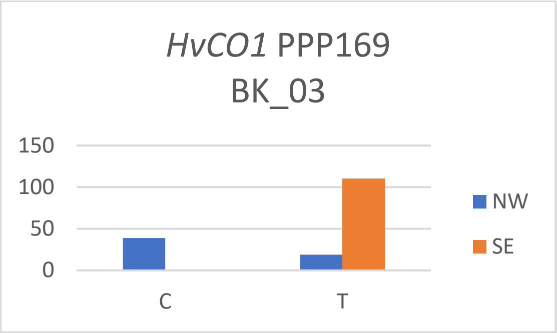

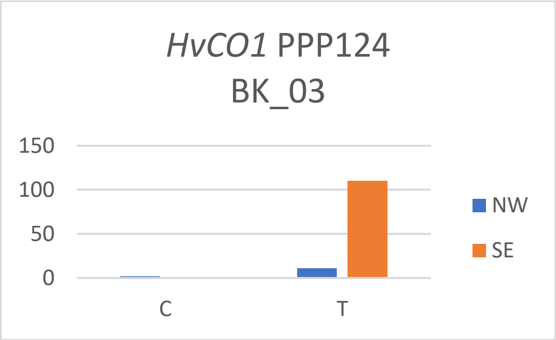

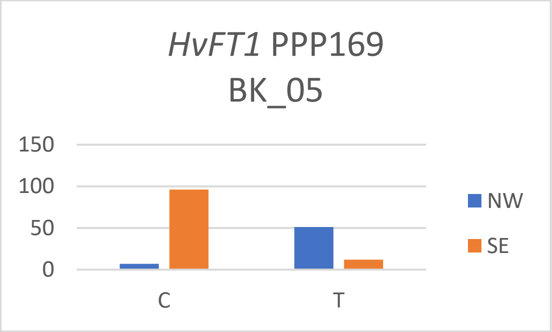

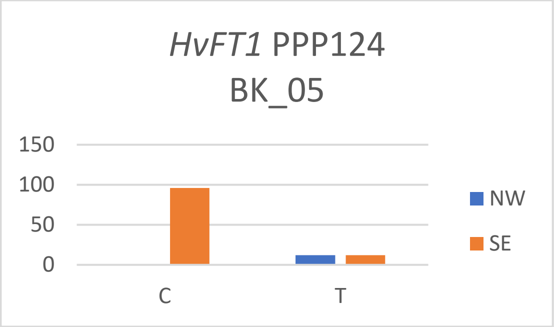

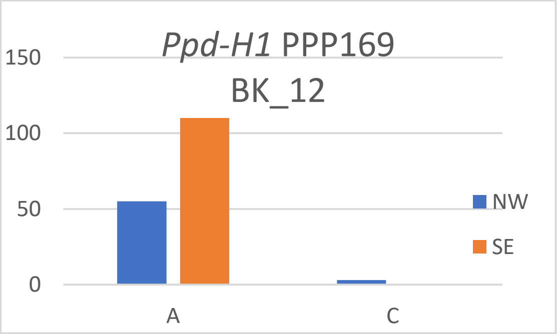

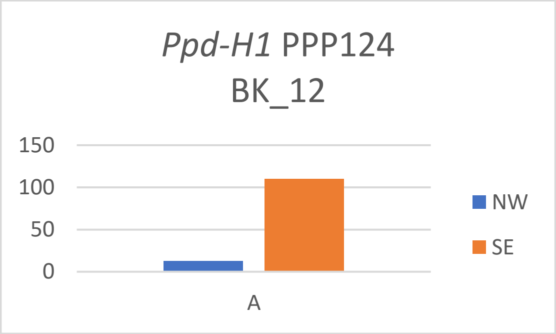

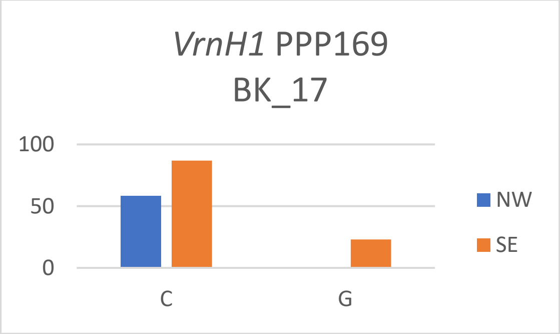

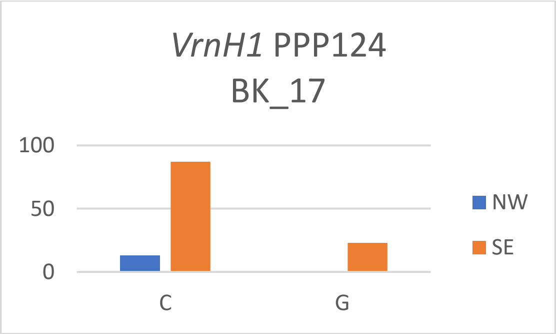

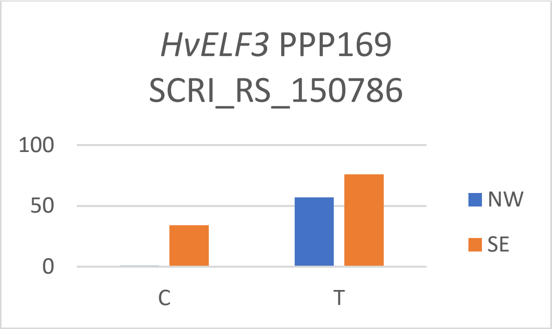
HvELF3 PPP169
SCRI_RS_150786
100
50
0
C
T
NW
SE

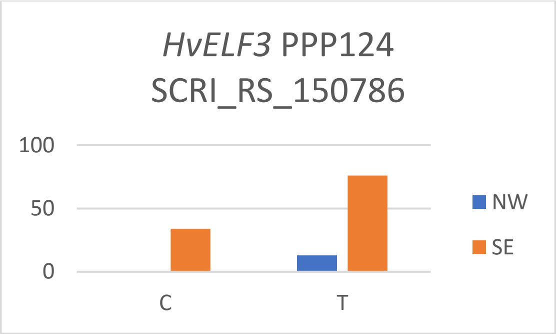
HvELF3 PPP124
SCRI_RS_150786
100
50
0
C
T
NW
SE

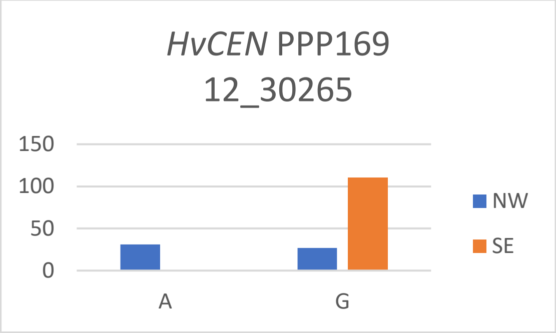
HvCEN PPP169
12_30265
150
100
50
0
A
G
NW
SE

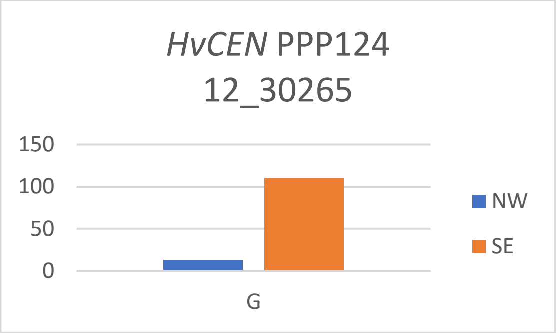
HvCEN PPP124
12_30265
150
100
50
0
G
NW
SE

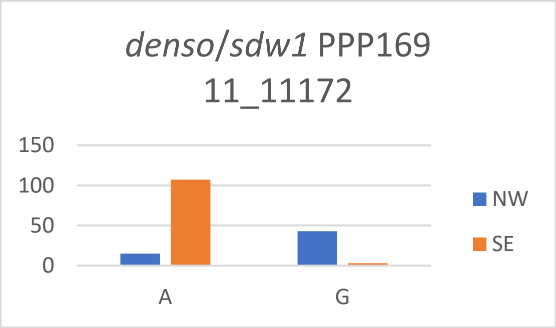
denso/sdw1 PPP169
11_11172
150
100
50
0
A
G
NW
SE

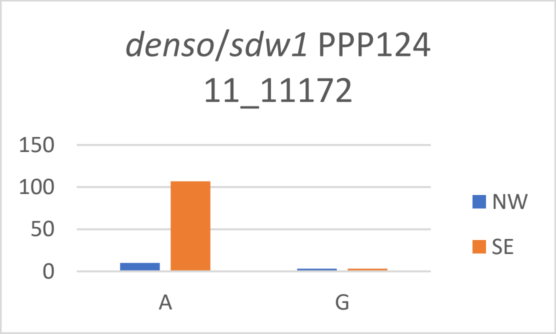
denso/sdw1 PPP124
11_11172
150
100
50
0
A
G
NW
SE
