# Supplementary material for: Identification of Ideal Allele Combinations for the Adaptation of Spring Barley to Northern Latitudes
Source: Front Plant Sci. 2019 May 3;10:542. doi: 10.3389/fpls.2019.00542 (PMC6510284; doi:10.3389/fpls.2019.00542)

Figure S3. Boxplots of allele combinations showing BLUE values for Ht34, HSHD, Sb and StL based on the most significant markers from GWAS analyses. The effect of each allele combination for combinations with at least five observations (lines) was calculated based on BLUE values and the significance of the effects was tested using the lm () function in R. * marks significant combinations ($p \leq 0.05$). Allele combinations followed by the same lower case letter do not differ statistically.

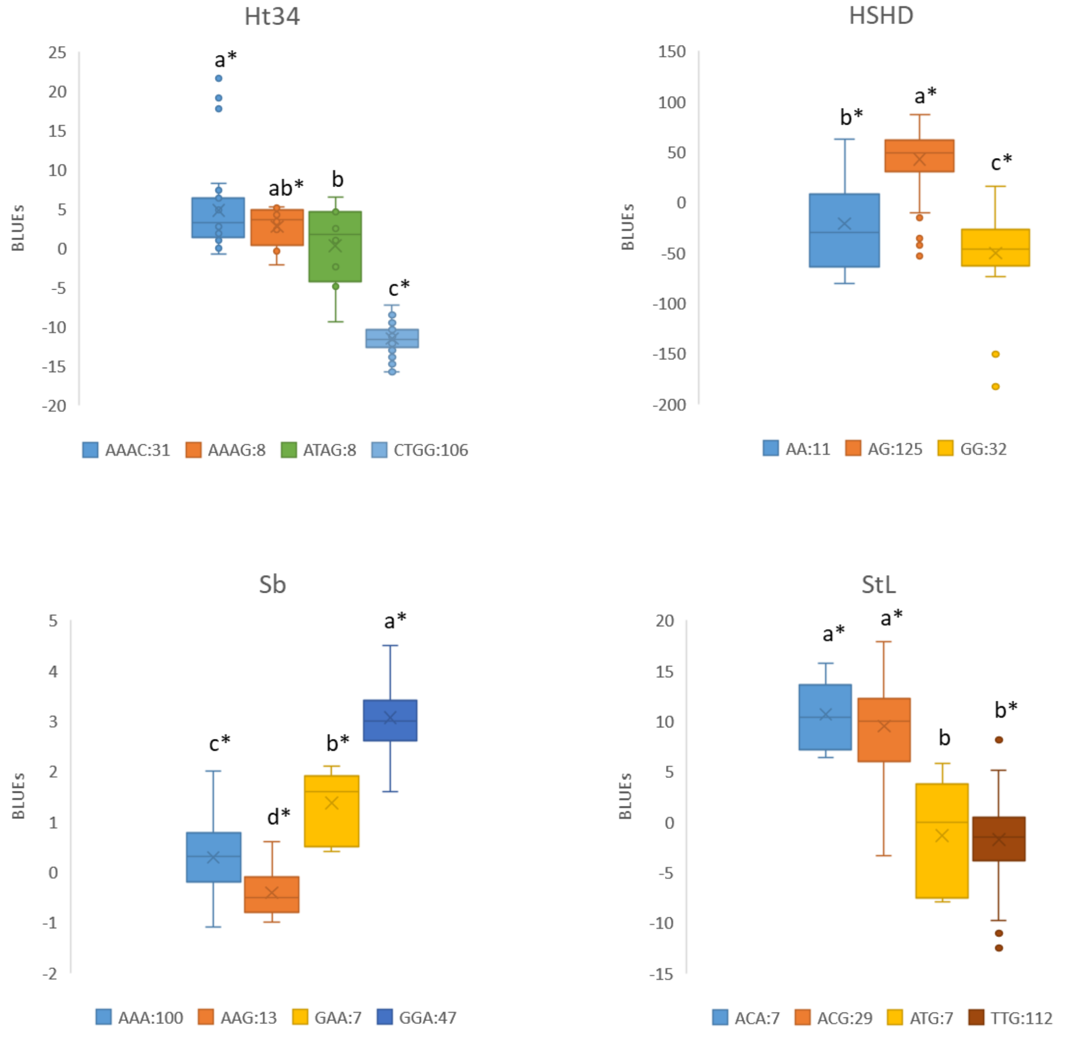

Supplement: Supplementary file 11 [file Data_Sheet_3.pdf]
